# TPP1 mutagenesis screens unravel shelterin interfaces and functions in hematopoiesis

**DOI:** 10.1172/jci.insight.138059

**Published:** 2021-05-10

**Authors:** Sherilyn Grill, Shilpa Padmanaban, Ann Friedman, Eric Perkey, Frederick Allen, Valerie M. Tesmer, Jennifer Chase, Rami Khoriaty, Catherine E. Keegan, Ivan Maillard, Jayakrishnan Nandakumar

**Affiliations:** 1 Department of Molecular, Cellular, and Developmental Biology,; 2 Life Sciences Institute,; 3 Department of Internal Medicine,; 4 Graduate Program in Cellular and Molecular Biology, and; 5 Medical Scientist Training Program, University of Michigan, Ann Arbor, Michigan, USA.; 6 Division of Hematology/Oncology, Department of Medicine, University of Pennsylvania Perelman School of Medicine, Philadelphia, Pennsylvania, USA.; 7 Department of Cell and Developmental Biology,; 8 Department of Pediatrics, and; 9 Department of Human Genetics, University of Michigan, Ann Arbor, Michigan, USA.; 10 Abramson Family Cancer Research Institute, University of Pennsylvania Perelman School of Medicine, Philadelphia, Pennsylvania, USA.

**Keywords:** Genetics, Hematology, Bone marrow, Cellular senescence, Telomeres

## Abstract

Telomerase catalyzes chromosome end replication in stem cells and other long-lived cells. Mutations in telomerase or telomere-related genes result in diseases known as telomeropathies. Telomerase is recruited to chromosome ends by the ACD/TPP1 protein (TPP1 hereafter), a component of the shelterin complex that protects chromosome ends from unwanted end joining. TPP1 facilitates end protection by binding shelterin proteins POT1 and TIN2. TPP1 variants have been associated with telomeropathies but remain poorly characterized in vivo. Disease variants and mutagenesis scans provide efficient avenues to interrogate the distinct physiological roles of TPP1. Here, we conduct mutagenesis in the TIN2- and POT1-binding domains of TPP1 to discover mutations that dissect TPP1’s functions. Our results extend current structural data to reveal that the TPP1-TIN2 interface is more extensive than previously thought and highlight the robustness of the POT1-TPP1 interface. Introduction of separation-of-function mutants alongside known TPP1 telomeropathy mutations in mouse hematopoietic stem cells (mHSCs) lacking endogenous TPP1 demonstrated a clear phenotypic demarcation. TIN2- and POT1-binding mutants were unable to rescue mHSC failure resulting from end deprotection. In contrast, TPP1 telomeropathy mutations sustained mHSC viability, consistent with their selectively impacting end replication. These results highlight the power of scanning mutagenesis in revealing structural interfaces and dissecting multifunctional genes.

## Graphical abstract

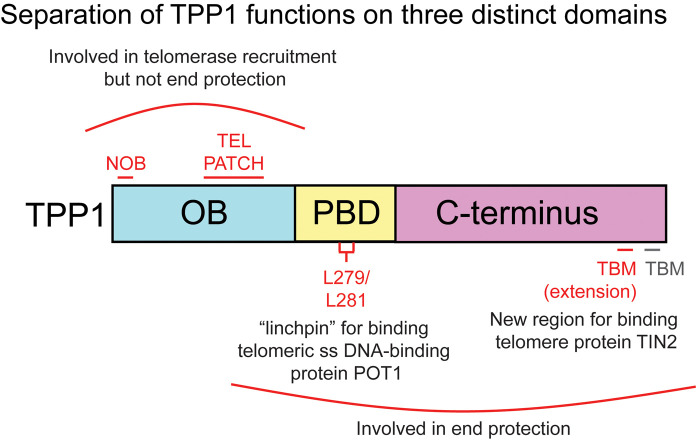

## Introduction

**Figure 1:**
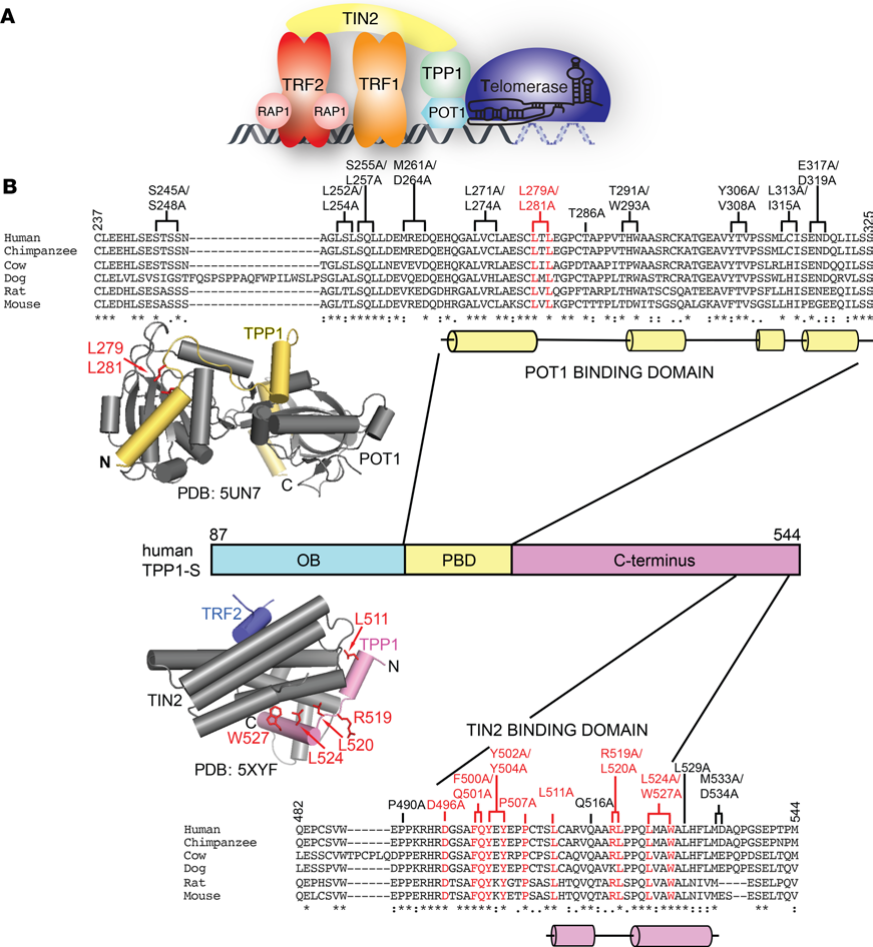
Screen to identify the TPP1 mutations that interfere with POT1 and TIN2 binding. (**A**) Schematic representation of the interactions between shelterin proteins and telomerase at chromosome ends. (**B**) Sequence alignment of the human TPP1 POT1- and TIN2-binding domains with indicated mammalian orthologs. Residues of human TPP1 that were mutated in this screen are shown above the alignment. TPP1 mutants defective in binding POT1 and TIN2 are highlighted in red. Brackets indicate 2 residues simultaneously mutated (double mutant). Asterisks, colons, and periods beneath the sequence lineups represent identical residues, strongly conserved residues, and weakly conserved residues, respectively, as described by the MUSCLE algorithm. Cylinders underneath the sequence alignment indicate α helices. The structure of the POT1 C-terminus bound to the TPP1-PBD (Protein Data Bank [PDB]: 5UN7) is shown above the TPP1 domain diagram with POT1 shown in gray and TPP1 shown in yellow. Structure of the TIN2_TRFH_-TPP1_TBM_-TRF2_TBM_ complex (PBD: 5XYF) is shown below the TPP1 domain diagram with TIN2_TRFH_ represented in gray, TRF2_TBM_ represented in purple, and TPP1_TBM_ represented in pink. TPP1 amino acids whose mutation resulted in POT1- and TIN2-binding defects are shown in red in the structures.

Telomeres are nucleoprotein complexes made up of a repetitive double-stranded (ds) DNA sequence (tandem GGTTAG repeats in mammals) that ends in a short G-rich single-stranded (ss) overhang and multiple copies of a 6-protein complex called shelterin (Figure 1A) (1). Telomeres are faced with and help overcome 2 problems: the end replication problem and the end protection problem, both of which can contribute to human disorders. The end replication problem results in the loss of DNA that occurs at the ends of chromosomes during DNA replication (2). If this loss is uncompensated, telomeres progressively shrink with each cell division until a critical limit is reached and cellular senescence is triggered. Telomerase is a unique ribonucleoprotein complex enzyme composed of a core protein, TERT, and an RNA subunit, TERC, that uses an internal RNA template to reverse-transcribe new telomeric DNA repeats at chromosome ends, helping counter the end replication problem (Figure 1A) (3–6). Consistent with this, telomerase is expressed in germline and somatic stem cells but is strictly repressed upon differentiation. Additionally, telomerase upregulation is characteristic of approximately 90% of all human cancers, highlighting telomerase’s ability to provide replicative immortality (7). While unregulated action of telomerase in somatic cells is a hallmark of cancer, germline mutations in genes coding for telomerase or proteins involved in telomere maintenance result in a myriad of diseases that are collectively termed telomeropathies. This wide spectrum of disorders is characterized by telomere dysfunction and severely short telomeres, often resulting from primary or secondary telomerase deficiency (8). A major vulnerability in telomeropathies is hematopoietic stem cell failure, as bone marrow failure is the major cause of morbidity and mortality in patients suffering from dyskeratosis congenita (DC), one of the most prominent telomeropathies (9–11). To date, germline mutations in up to 14 genes have been reported to underlie congenital telomeropathies (12, 13).

The end protection problem describes the propensity of natural chromosome ends to participate in unwanted recombination or end-to-end fusion events. The 6-protein complex shelterin (TRF1, TRF2, Rap1, TIN2, ACD/TPP1, and POT1) prevents the activation of the ATM and ATR kinases at telomeres to protect chromosome ends from being recognized as sites of DNA damage (Figure 1A) (1). A growing number of mutations in genes coding for shelterin proteins have also been reported in human disease (11).

Within shelterin, TRF1 and TRF2 bind the ds telomeric DNA (Figure 1A), with TRF2 functioning to block the ATM kinase–mediated DNA damage response (14, 15). The shelterin protein POT1 carries out end protection by binding specifically to the G-rich ss overhang, thereby excluding the ssDNA-binding protein RPA and preventing activation of ATR kinase at telomeres (16–19). The shelterin protein ACD/TPP1 (adrenocortical dysplasia homolog/TINT1-PTOP-PIP1, hereafter referred to as TPP1; human gene name: *ACD*; mouse gene name: *Acd*; HUGO Gene Nomenclature Committee Symbol: ACD) binds POT1 and increases its affinity for ss telomeric DNA (20–23). Structural studies with the C-terminal domain of POT1 in complex with the POT1 binding domain (PBD) of TPP1 provide important insights into the POT1-TPP1 protein interface (24, 25) (Figure 1B). Consistent with the role of this interface in ensuring genome stability via chromosome end protection, mutations within the TPP1-binding region of POT1 are associated with chronic lymphocytic leukemia and familial melanomas (26–28). Along with enhancing POT1’s affinity for DNA, TPP1 recruits POT1 to telomeres (29, 30). Consistent with this, depletion of TPP1 results in a robust ATR response (29, 30). Homozygosity for the hypomorphic *acd* allele leads to defective hematopoietic stem cell function in a mouse transplantation model, while acute global *Acd* loss induces cell cycle arrest of hematopoietic progenitors and rapid hematopoietic failure (31).

TIN2 plays a central role in the shelterin complex by bridging the POT1-TPP1 heterodimer at the ss telomeric DNA to TRF1 and TRF2 at the ds telomeric DNA (Figure 1A) (32, 33). While TRF1 is required for TIN2 recruitment to telomeres (34), TIN2 recruits POT1-TPP1 to telomeres by directly interacting with the C-terminus of TPP1 (22, 35) (Figure 1B). Interestingly, TPP1 binding also promotes TIN2’s affinity for TRF2 (36, 37). A structure of a ternary protein/peptide complex shows how the TIN2_TRFH_ domain (TRF Homology domain originally described in TRF1 and TRF2 proteins) simultaneously binds a TPP1 TIN2-binding motif-containing peptide (TPP1_TBM_; aa 510–544) and a TRF2 TIN2-binding motif-containing peptide (TRF2_TBM_; aa 350–366) (36) (Figure 1B). Because the TPP1_TBM_ and TRF2_TBM_ peptides do not directly interact with each other in this structure, current models fail to provide an obvious mechanism for the observed cooperativity.

TPP1 is at the crossroads of both end protection and end replication because it not only binds POT1 and TIN2 to protect chromosome ends but also binds telomerase to recruit it to telomeres (Figure 1A) (20, 38). Telomerase is recruited to telomeres via an interaction between the catalytic protein subunit of telomerase TERT and the N-terminal OB domain of TPP1 (38, 39). Mutagenesis studies on TERT have revealed the TEN domain and IFD as 2 regions that are critical for interacting with TPP1 (40–44). Site-directed mutagenesis of the OB domain of TPP1 revealed 2 regions, the TEL patch and NOB, as being critical for TPP1’s interaction with TERT (40, 41, 45, 46). TEL patch and NOB mutations represent true separation-of-function mutations in the TPP1 OB domain, as they abrogate telomerase recruitment to telomeres but do not affect TPP1’s ability to bind POT1 or TIN2 (45, 47). Mutations in the TEL patch have been observed in individuals suffering from telomeropathies. A severe form of DC known as Hoyeraal-Hreidarsson syndrome (HH) results from deletion of K170, an amino acid that is adjacent to residues that form the TPP1 TEL patch (47, 48). The same mutation was also seen in an unrelated patient with aplastic anemia (49). Interestingly, the proband with HH was a compound heterozygote for *ACD* variants, as the allele lacking the K170 deletion coded for a variant (P491T) in the TIN2-binding region of TPP1. This mutation moderately reduced binding of TPP1 to TIN2 in vitro, but its impact on telomere maintenance in vivo remains unknown (47). Like the TEL patch, mutations in TPP1 NOB have also been associated with individuals presenting with telomeropathy-like symptoms (50).

Based on our current understanding, the TPP1 protein harbors 3 interfaces that contribute to telomere function by operating through either end protection or telomerase regulation (Figure 1B). Previous in vivo studies fall short of separating end protection from end replication phenotypes because they have involved full knockout, hypomorphic alleles, or broad domain deletions of TPP1. Conversely, technical limitations have restricted structural studies to minimal domains/peptides of TPP1 and other shelterin proteins, preventing investigations into putative crosstalk between the different TPP1 domains and their associated functions. We conducted a site-directed mutagenesis screen in the TIN2- and POT1-binding regions of full-length TPP1 (Figure 1B) to map new interacting regions within these proteins, discover separation-of-function mutations that disrupt only 1 binding interface of TPP1, and compare the physiological consequences of these mutations with telomeropathy-associated mutations of *ACD* in mouse hematopoietic stem cells. Together with our prior site-directed mutagenesis studies in the TPP1 OB domain, this study provides a more complete structure-function map of the human TPP1 protein at single–amino acid resolution.

## Results

### Site-directed mutagenesis of TPP1 reveals an extended TPP1-TIN2 interaction interface.

**Figure 2:**
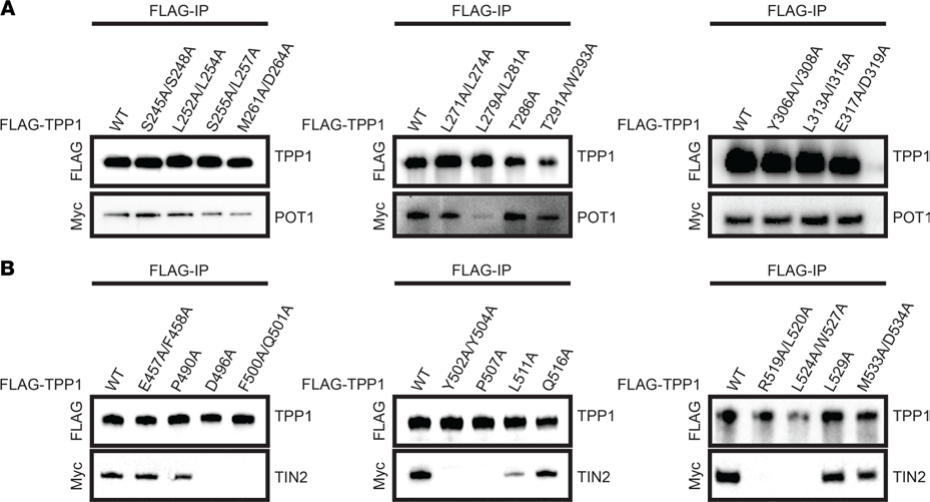
Mutations in the TPP1_PBD_ and the TPP1 C-terminus disrupt POT1 or TIN2 binding. (**A**) Pulldown of transiently expressed FLAG-TPP1_PBD_ mutants on anti-FLAG–conjugated beads with Myc-POT1. (**B**) Pulldown of transiently expressed FLAG-TPP1 C-terminus mutants on anti-FLAG–conjugated beads with Myc-TIN2. These data are representative of at least 2 independent transfections and co-IPs (*n* = 2).

To unravel distinct molecular functions of TPP1 in the shelterin complex, we designed a site-directed alanine scanning mutagenesis screen focusing on conserved residues predicted to lie on the surface of the human TPP1 protein (Figure 1B). The human TPP1-S isoform, initiating at Met87, was used in this study as it is conserved across mammals and sufficient for all known functions of TPP1 in somatic cells (51). We engineered 1 single–amino acid mutant and 10 double mutants in TPP1’s POT1-binding domain (TPP1-PBD; Figure 2A), as well as 6 single–amino acid mutants and 6 double mutants in the C-terminus of TPP1 (TPP1 TIN2-binding region; Figure 2B, mutant E457A/F458A not shown in Figure 1B). We conducted coimmunoprecipitation experiments with transiently coexpressed FLAG-tagged TPP1 and Myc-tagged POT1 or Myc-tagged TIN2 to determine how these mutants affected TPP1 binding. Double mutant (L279A/L281A) in the TPP1-PBD showed reduced POT1 binding (Figure 2A, center). This is consistent with published structures (24, 25), which show residues L279 and L281 at the end of the TPP1 PBD α helix 1 buried in a hydrophobic pocket on the surface of the POT1 Holliday junction resolvase-like domain (Figure 1B). However, POT1 coimmunoprecipitated with TPP1 in the presence of all other introduced mutations (Figure 2A), even those residues that appear to contribute to the interaction based on the crystal structures. These findings highlight the extensiveness of this interface as well as corroborate the notion that POT1-TPP1 is an obligate heterodimer at mammalian chromosome ends. In summary, mutagenesis of the POT1-binding domain of TPP1 reveals a robust interface that is generally resistant to mutations and identifies L279/L281 as the “linchpin” of the POT1-TPP1 interface.

Although both the POT1-TPP1 and TPP1-TIN2 interfaces proved to be extensive, the latter interface was easier to disrupt. Six TPP1 mutants completely abrogated binding to TIN2 (D496A, F500A/Q501A, Y502A/Y504A, P507A, R519A/L520A, and L524A/W527A), and 1 mutant showed reduced binding (L511A) (Figure 2B). The residues mutated in R519A/L520A and L524/W527A are involved in contacting TIN2 in the published structure of the TIN2_TRFH_ domain bound to the TPP1_TBM_ (Figure 1B) (36). R520, L524, and W527 together form part of an extended hydrophobic interface between the TPP1_TBM_ and the TIN2_TRFH_ domain. Interestingly, residues D496, F500, Q501, Y502, Y504, and P507 are part of the original description of the TIN2-binding region of TPP1 (22) but were not included in the definition of TPP1_TBM_ used in the determination of the TPP1-TIN2 interface structure. Thus, the structural basis of how these residues bind TIN2 remains unknown. Our coimmunoprecipitation analysis indicates that these residues are as critical to TIN2 binding as TPP1_TBM_ residues (Figure 2B). This suggests that the TPP1-TIN2 interface extends beyond the TPP1_TBM_. We call this newly identified region the TBM extension, or TPP1_TBM-ext_. Our mutagenesis studies on the TIN2-binding region of TPP1 suggest that the TPP1-TIN2 interface is more elaborate than previously appreciated.

### TPP1_TBM-ext_ is important for enhancing the TIN2-TRF2 interaction.

**Figure 3:**
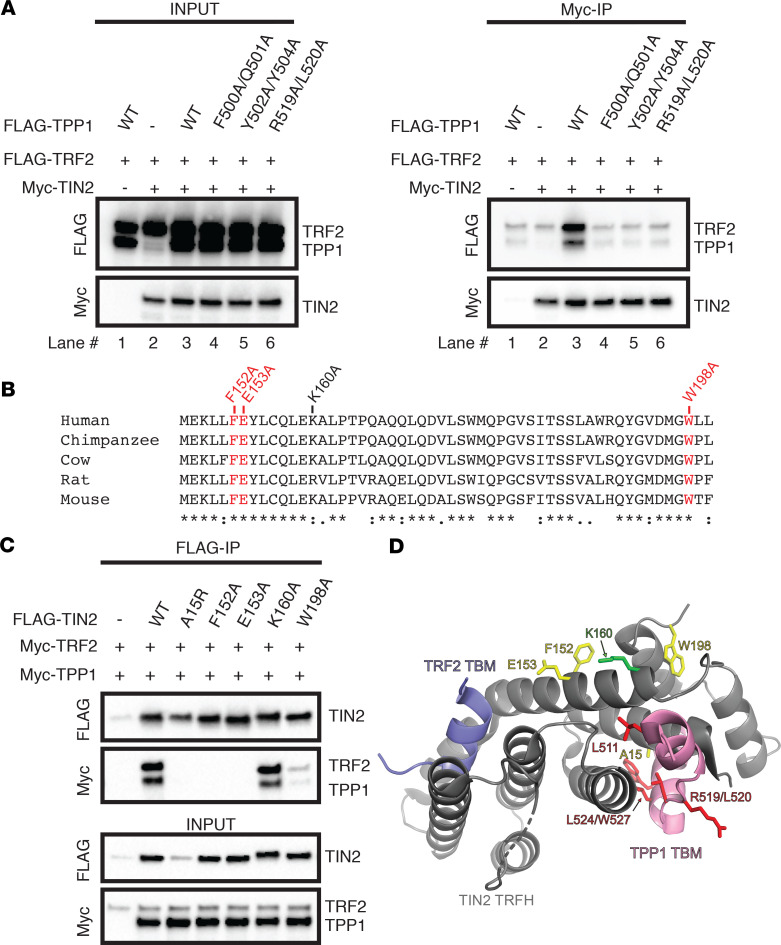
Mutations in the TPP1_TBM_, TPP1_TBM-ext_, or TIN2 disrupt cooperativity of the TPP1-TIN2-TRF2 interaction. (**A**) Anti-Myc antibody–bound Myc-TIN2 was pulled down on protein A/G agarose beads with transiently expressed FLAG-TRF2 and indicated FLAG-TPP1 construct. (**B**) Sequence conservation of a portion of the TIN2 TRFH domain. TIN2 residues examined in this study are labeled above the sequence alignment, with red denoting amino acids whose mutation impaired the TPP1-TIN2 interaction and black denoting amino acids whose mutation did not affect TPP1-TIN2 binding. Asterisks, colons, and periods beneath the sequence lineups represent identical residues, strongly conserved residues, and weakly conserved residues, respectively, as described by the MUSCLE algorithm. (**C**) Pulldown of indicated FLAG-TIN2 construct on anti-FLAG–conjugated beads with WT Myc-TRF2 and Myc-TPP1. (**D**) Structure of the TIN2_TRFH_ (gray)-TPP1_TBM_ (pink)- TRF2_TBM_ (lilac) complex (PDB: 5XYF, ref. 36). TIN2 residues whose mutation impacts binding to TPP1 and that likely contact the TPP1_TBM-ext_ are shown in yellow, TIN2_K160A_ is shown in green, and TPP1_TBM_ mutants are shown in red. Data in **A** and **C** are representative of at least 5 independent transfections and coimmunoprecipitations (co-IPs) (*n* = 5).

TPP1 has previously been shown to promote the interaction between TIN2 and TRF2 (36, 52). We asked if mutations in the TPP1_TBM-ext_ prevent this cooperativity. Coimmunoprecipitation experiments revealed that Myc-tagged TIN2 efficiently pulled down transiently coexpressed WT TPP1 and TRF2 (right panel; Figure 3A, lane 3). In contrast, although some TRF2 nonspecifically bound the beads, TIN2 was unable to efficiently pull down TRF2 in the absence of TPP1 (right panel; Figure 3A, compare lanes 1–3). Coexpression of TPP1_TBM_ mutant R519A/L520A was unable to rescue TRF2 binding, as TIN2 did not pull down TPP1 R519A/L520A or WT TRF2 (Figure 3A, lane 6). Importantly, mutants in the newly identified TPP1_TBM-ext_ also blocked this cooperativity as TIN2 did not pull down WT TRF2 with either TPP1 F500A/Q501A or TPP1 Y502A/Y504A (Figure 3A, lanes 4 and 5). Together, these data suggest that the TPP1_TBM-ext_ is important for promoting the TIN2-TRF2 interaction.

### Extending the definition of the TPP1-binding region of TIN2_TRFH_.

We next asked how the TPP1_TBM-ext_ interacts with the TRFH domain of TIN2. We hypothesized that the TPP1_TBM-ext_ binds a region on the TIN2_TRFH_ domain that is adjacent to the TPP1_TBM_ footprint captured in the crystal structure. To test this hypothesis and identify TIN2 residues that are important for binding the TPP1_TBM-ext_, we conducted site-directed mutagenesis of 4 conserved and surface-exposed residues in TIN2_TRFH_ that are proximal to the N-terminus of the TPP1_TBM_ peptide based on the crystal structure of the TPP1_TBM_-TIN2_TRFH_-TRF2_TBM_ complex (Figure 3, B and D). We asked how mutation of these residues to alanine affected binding of TIN2 to WT TPP1 and TRF2. As expected, the previously characterized TIN2_A15R_ mutation, which abrogates the interaction between TIN2 and TPP1, was unable to pull down WT TPP1 or TRF2 (Figure 3C). Importantly, mutation of either TIN2 F152 or TIN2 E153 to alanine resulted in a complete loss of TPP1 and TRF2 binding (Figure 3C). These residues likely contact the TPP1_TBM-ext_, as they reside about 20 Å from the N-terminus of the TPP1_TBM_ (Figure 3D). In contrast, mutation of residue W198 of TIN2_TRFH_ resulted in a partial loss of TPP1 and TRF2 binding, while our data suggest that K160 is not important for the interaction with either TPP1 or TRF2 (Figure 3C). Together with the discovery of TPP1_TBM-ext_, these TIN2 mutagenesis data expand our understanding of the TIN2-TPP1 interface.

We asked how these separation-of-function mutations impact TPP1 and TIN2 localization to telomeric DNA in cells. TRF1 interacts with TIN2 in a region that resides outside of the TIN2-TPP1 binding domain. Indeed, all TIN2 mutants analyzed colocalized with telomeric DNA, suggesting that these mutations do not affect the TIN2-TRF1 association or recruitment to the telomere (Supplemental Figure 1A; supplemental material available online with this article; https://doi.org/10.1172/jci.insight.138059DS1). Similarly, TPP1 PBD mutant L279A/L281A successfully localized to telomeric DNA, consistent with this mutation not perturbing the TPP1-TIN2 interaction that is necessary for TPP1 recruitment to telomeres (Supplemental Figure 1B). In stark contrast, both TPP1_TBM_ and TPP1_TBM-ext_ mutants did not colocalize with telomeric DNA, and were largely nuclear excluded, consistent with the loss of TIN2 binding in these mutants (Supplemental Figure 1B). Together, these data support the importance of the TPP1_TBM-ext_ for TIN2 binding in vivo and highlight how the identified mutations selectively impact only the intended interface.

### Separation-of-function mutations that disrupt TPP1 interactions within shelterin result in acute hematopoietic failure.

**Figure 4:**
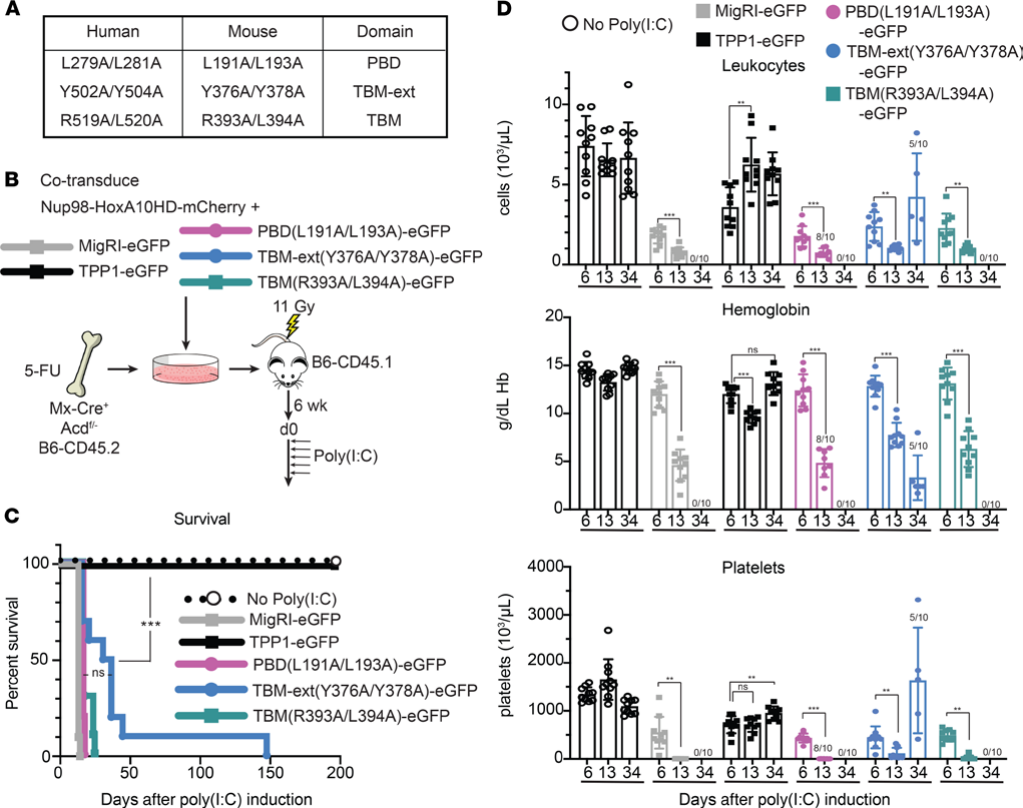
Hematopoietic progenitors with mutations in TPP1_PBD_, TPP1_TBM_, or TPP1_TBM-ext_ cannot rescue mouse hematopoiesis after loss of endogenous *Acd*. (**A**) Schematic of representative TPP1_PBD_ (L191A/L193A), TPP1_TBM_ (Y376A/Y378A), or TPP1_TBM-ext_ (R393A/L394A) mutations in mice, with their equivalent in humans. (**B**) Experimental scheme. BM was harvested from 5-fluorouracil–treated (5-FU–treated) *Mx-Cre*^+^ *Acd^fl/–^* B6-CD45.2 mice and retrovirally cotransduced with NUP98-HOXA10HD-mCherry and a TPP1 rescue construct expressing an EGFP reporter (vs. EGFP only). Transduced BM progenitors were transplanted into lethally irradiated congenic B6-CD45.1 recipients. Six weeks later, endogenous *Acd* was inactivated via poly(I:C) administration to induce *Mx-Cre* expression. (**C**) Survival and (**D**) complete blood counts of transplanted mice at days 6, 13, and 34. *n* = 10 per group; remaining numbers of mice are noted in **D**. ***P < 0.001 by log-rank Mantel-Cox test (**C**). **P < 0.01, ***P < 0.001 by 2-way ANOVA with post hoc Tukey’s test to assess differences in means in **D**. Mean and 1 SD reported.

To evaluate the impact of TPP1 interactions with TIN2 and POT1 in vivo, we turned to hematopoiesis as a relevant organ system prominently affected in human telomeropathies. Specifically, we used the maintenance of mouse hematopoietic stem cells (HSCs) as a sensitive in vivo readout of shelterin’s end protection functions. We previously observed rapid HSC loss and hematopoietic failure upon acute *Acd* inactivation and loss of the TPP1 protein in bone marrow (BM) hematopoietic stem and progenitor cells (31). Upon *Acd* inactivation (in *Acd^fl/fl^* mice; The Jackson Laboratory stock 021983), we previously showed that HSCs underwent cell cycle arrest and induction of p53 target genes within 2 days after induction of *Acd* loss, followed by evidence of chromosomal instability and end fusion events consistent with an end deprotection phenotype. Building on this observation, we designed a system to replace endogenous TPP1 with retrovirally expressed WT or mutant TPP1 variants to generate an in vivo structure-function assay in mouse HSCs (Figure 4, A and B). Based on our in vitro observations (Figure 2), we selected the L279A/L281A TPP1 mutation to disrupt the TPP1-POT1 interface, as well as the Y502A/Y504A and R519A/L520A TPP1 mutations to impair TIN2’s interaction with the TPP1_TBM-ext_ or TPP1_TBM_, respectively. We cloned the mouse equivalents of these mutants (L191A/L193A, Y376A/Y378A, R393A/L394A) in mouse stem cell virus–based (MSCV-based) retroviral vectors (Figure 4A). We then harvested BM cells from 5-FU–treated *Mx-Cre^+^ Acd^fl/–^* C57BL/6(B6)-CD45.2 mice and cotransduced BM hematopoietic stem and progenitor cells with an mCherry-tagged retrovirus expressing NUP98-HOXA10HD, as well as an EGFP-tagged retrovirus expressing EGFP (MigR1-EGFP, negative control), or EGFP plus WT TPP1 (positive control), versus EGFP and TPP1 mutants of interest (Figure 4B). NUP98-HOXA10HD was selected for its capacity to expand HSCs in vitro and in vivo without inducing transformation (53–55), which allowed us to generate robust HSC grafts in which the function of TPP1 variants could be assessed. Transduced BM cells were used to reconstitute lethally irradiated congenic B6-CD45.1 recipients, with stable engraftment of EGFP^+^ cells over 6 weeks for all constructs (ruling out a dominant-negative activity of WT TPP1 or TPP1 mutants). We then induced *Mx-Cre* expression with poly(I:C) administration to inactivate endogenous *Acd*. Thus, we could assess the capacity of WT TPP1 or individual TPP1 mutants to rescue steady-state hematopoiesis and avoid the acute effects of *Acd* loss that we previously documented (31).

Within 10–20 days after poly(I:C) administration and *Acd* inactivation, all recipients of the MigR1-EGFP–transduced HSCs showed rapid lethality as expected, while recipients of HSCs expressing WT TPP1 were fully rescued (Figure 4C). In contrast, expression of the L191A/L193A, Y376A/Y378A, and R393A/L394A TPP1 mutants did not prevent lethality. The TPP1_PBD_ mutant L191A/L193A and the TPP1_TBM_ mutant R393A/L394A failed to prevent lethality similar to the empty vector control, while the TPP1_TBM-ext_ mutant (Y376A/Y378A) allowed for slightly prolonged survival compared with empty vector in a fraction of mice. Tracking of the complete blood counts at day 6, 13, and 34 after poly(I:C) showed preservation of blood leukocytes, platelets, and hemoglobin levels in recipients of WT TPP1-transduced HSCs (Figure 4D). In contrast, profound progressive pancytopenia was observed in the majority of mice reconstituted with HSCs expressing EGFP only, or EGFP and L191A/L193A, Y376A/Y378A, and R393A/L394A TPP1 mutants. Thus, targeted molecular interference with mutations that disrupt the interaction of TPP1 with POT1 or TIN2 failed to rescue HSC function in vivo.

**Figure 5:**
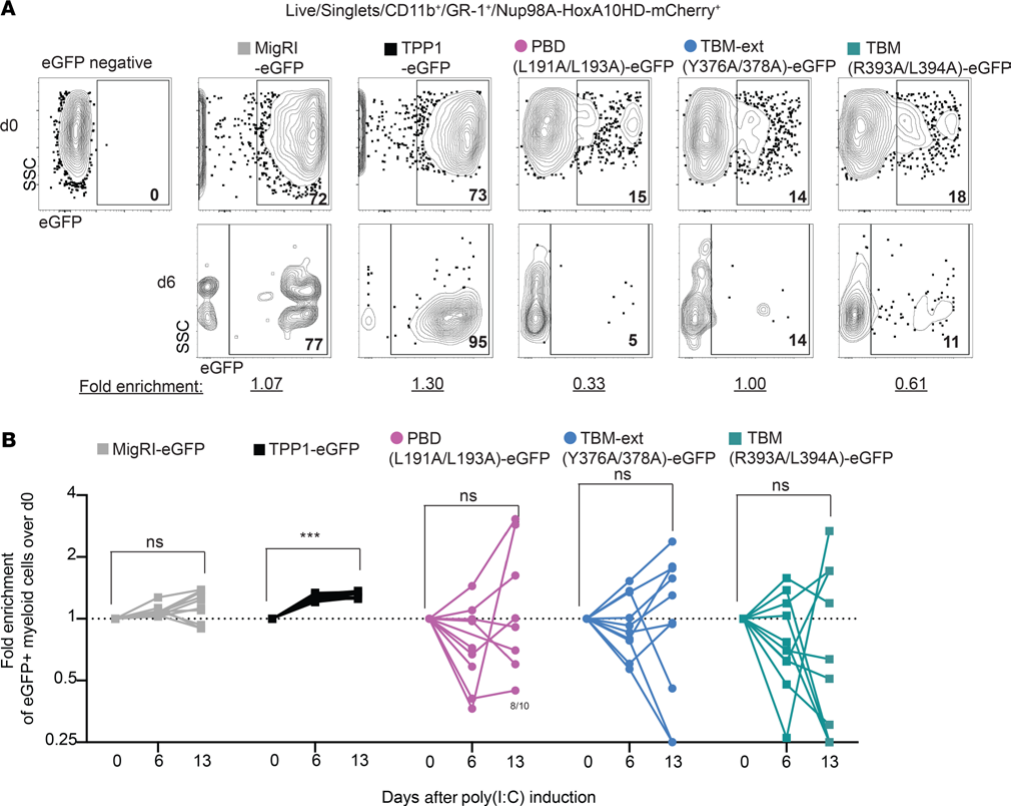
Mutations in the TPP1_PBD_, TPP1_TBM_, or TPP1_TBM-ext_ do not confer a selective advantage over *Acd*-null progenitors in vivo. (**A**) Donor-derived NUP98-HOXA10HD-mCherry^+^ CD11b^+^Gr-1^+^ myeloid cells were assessed by flow cytometric analysis of EGFP before poly(I:C)-induced loss of endogenous *Acd* (d0) or at day 6 after poly(I:C) induction. EGFP reports expression of WT TPP1 versus TPP1 mutants versus EGFP only control (MigR1). (**B**) Relative enrichment of EGFP^+^ myeloid cells at day 6 and 13 is noted and summarized. ***P < 0.001 by 2-way ANOVA with post hoc Tukey’s test to assess differences in means (**B**). *n* = 10 per group.

To further substantiate these observations, we assessed CD45.2/CD45.1 chimerism and the percentage of CD45.2^+^mCherry^+^ donor-derived cells expressing EGFP before and after poly(I:C). Before poly(I:C) administration, blood CD11b^+^Gr1^+^ myeloid cells showed near-complete replacement with CD45.2^+^mCherry^+^ cells (>95%), consistent with the capacity of NUP98-HOXA10HD to expand HSCs as described (53–55). This generated a very robust graft that was able to outcompete residual host CD45.1^+^ progenitors. Among NUP98-HOXA10HD-mCherry^+^ blood myeloid cells, we measured the percentage of EGFP^+^ cells (which varied based on viral titers and transduction efficiency) and tracked this percentage in individual mice on days 6, 13, and 34 after poly(I:C) to detect functional selection of transduced cells after loss of endogenous *Acd* (Figure 5 and Supplemental Figure 2). Marked enrichment for EGFP^+^ myeloid cells was already apparent at day 6 in recipients of HSCs transduced with WT TPP1 (Figure 5A and Supplemental Figure 2) and approached 100% at later time points (Figure 5B and Supplemental Figure 2). In contrast, we observed no consistent change in the percentage of EGFP over time in recipients of EGFP-transduced HSCs, or in recipients of HSCs transduced with L191A/L193A, Y376A/Y378A, or R393A/L394A TPP1 mutants (Figure 5, A and B, and Supplemental Figure 2, A–C). Of note, the lower percentage of basal EGFP expression in L191A/L193A, Y376A/Y378A, or R393A/L394A groups as compared with EGFP or TPP1 groups was consistent with the use of lower viral titers; however, subsequent changes in GFP expression appeared random, presumably based on the selection of rare GFP^–^ or GFP^+^ progenitors escaping endogenous *Acd* inactivation (Figure 5B). At the termination of the experiment, the only surviving mice were recipients of WT TPP1, with a majority of the mice showing close to 100% EGFP expression among BM hematopoietic stem and progenitor cells (Supplemental Figure 2, D and E).

Of note, we also analyzed EGFP expression among NUP98-HOXA10HD–expressing mCherry^+^ cells at days 7 and 10 of the initial ex vivo culture and in vivo among blood myeloid cells 6 weeks after transplantation but before poly(I:C)-induced *Acd* excision (Supplemental Figure 3A). The relative percentage of EGFP expression remained stable across all experimental groups or even increased slightly in some (Supplemental Figure 3, A and B). These findings are consistent with the lack of a significant dominant-negative effect of mutant TPP1 constructs. Instead, expression of L191A/L193A, Y376A/Y378A, or R393A/L394A TPP1 mutants in mouse HSCs did not confer a detectable selective advantage after endogenous *Acd* loss (Figure 5), suggesting that these mutants were incapable of rescuing TPP1’s end protection function in vivo.

### Separation-of-function mutations that disrupt TPP1 interactions with other shelterin proteins cause a DNA damage response at telomeres.

**Figure 6:**
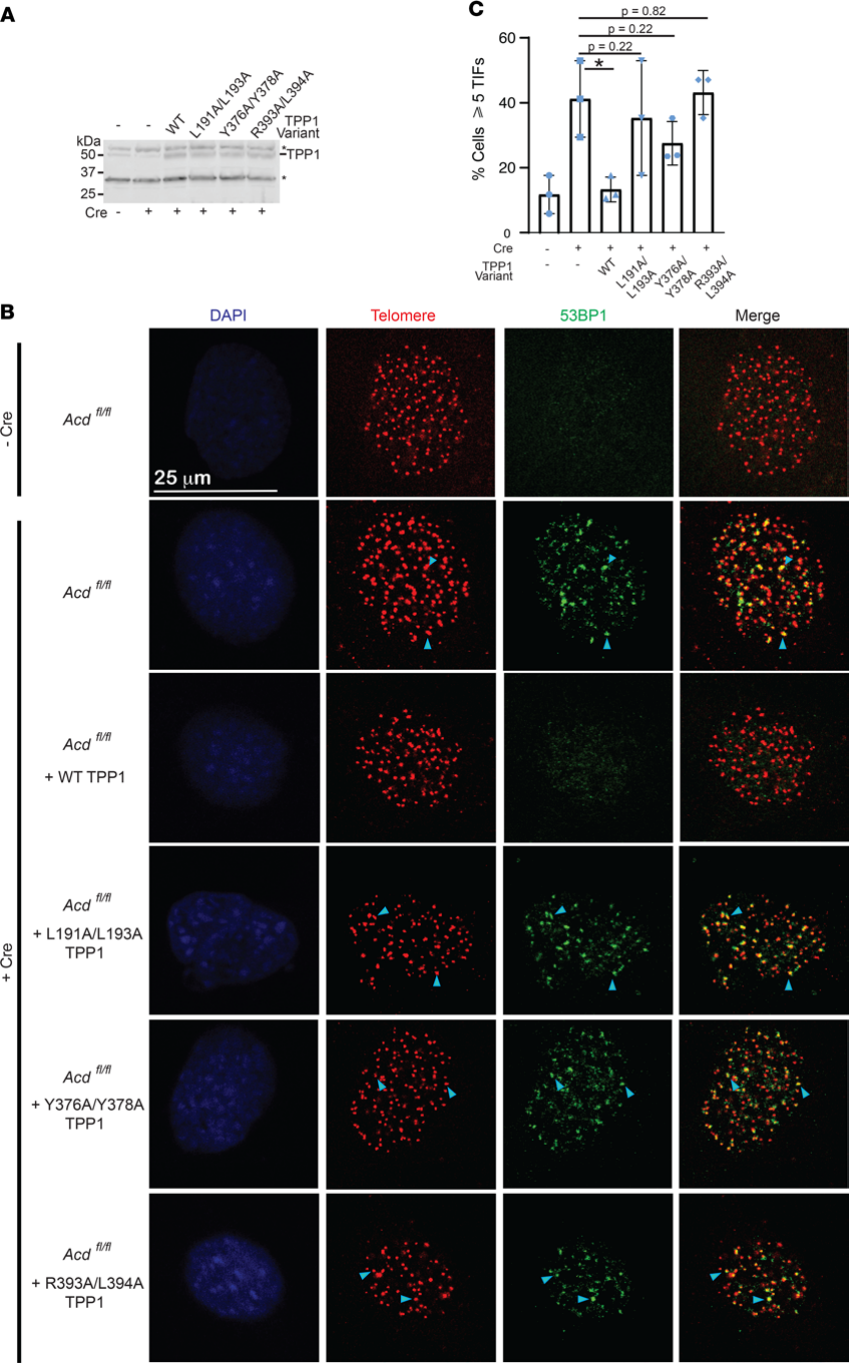
TPP1 mutants fail to rescue TPP1-KO cells from a DNA damage response at chromosome ends. (**A**) Immunoblot analysis of the indicated *Acd^fl/fl^* MEF cell lines showing reduction of TPP1 protein levels 72 hours after Cre-ER activation with 4-hydroxytamoxifen (4-OHT) and a uniform rescue of protein levels upon infection with indicated TPP1 WT or mutant lentiviruses. Asterisk indicates nonspecific bands detected by the antibody that serve as loading controls. *n* = 1. (**B**) TIF analysis was performed on the cell lines described in **A** using peptide nucleic acid–FISH for telomeres (red) and immunofluorescence for 53BP1 (green). DAPI was used to stain the nucleus (blue). Appearance of orange foci in the “Merge” panel indicates TIFs. Arrowheads point to 2 representative TIFs in the panel. (**C**) Quantitation of TIF data of which **B** is representative. Mean and SD for *n* = 3 sets of images (each set containing 15–20 cells) are plotted for the indicated cell lines. *P ≤ 0.05 using 2-tailed Student’s *t* test.

To further evaluate the molecular mechanism underlying the failure of TPP1 mutants to rescue the function of endogenous TPP1, we probed for DNA damage response markers at telomeres using telomere dysfunction induced foci (TIF) analysis. To that end, *Acd^fl/fl^* mouse embryonic fibroblasts (MEFs) (56) were conditionally knocked out for TPP1 using the Cre-*LoxP* recombination system, then subsequently transduced with either WT or mutant mouse TPP1-encoding lentiviruses. Loss of TPP1 protein expression upon addition of Cre and reconstituted expression of WT and mutant TPP1 was confirmed using immunoblotting of the MEF lysates (Figure 6A). MEFs conditionally knocked out for TPP1 resulted in TIF formation in 40% of cells examined, while reconstitution with WT TPP1 significantly suppressed this TIF response at telomeres, resulting in only 13% of cells with 53BP1 at telomeres (Figure 6, B and C). Consistent with the disruption of the POT1-TPP1 interaction, TPP1 L191A/L193A was not proficient in rescuing the TIF phenotype of the TPP1-KO cells (Figure 6, B and C). Additionally, TPP1 mutants that disrupted TIN2 binding either in TBM (TPP1 R393A/L394A) or TBM-ext (TPP1 Y376A/Y378A) failed to significantly reduce the TIF response, suggesting that both the TPP1_TBM_ and TPP1_TBM-ext_ are necessary for chromosome end protection (Figure 6, B and C). As TPP1_TBM_ and TPP1_TBM-ext_ mutants fail to localize to telomeric DNA (Supplemental Figure 1B), they likely induce TIF formation by binding endogenous POT1 and sequestering it away from the ssDNA overhang at chromosome ends. Together, these data indicate that the separation-of-function TPP1 mutants induce a DNA damage response at telomeres and that the TPP1_TBM-ext_, in addition to the TPP1_PBD_ and TPP1_TBM_, is essential for repressing DNA damage signaling at chromosome ends.

### Telomeropathic mutations in TPP1 do not cause immediate hematopoietic defects.

**Figure 7:**
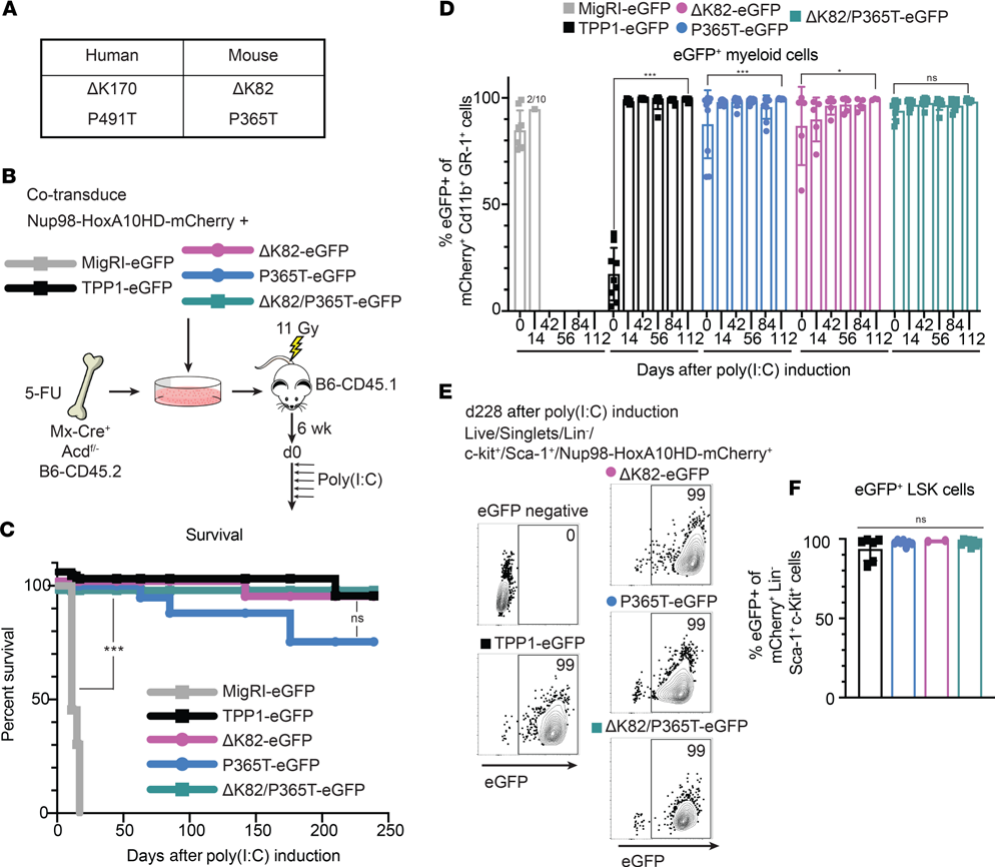
TPP1 mutants from human patients with telomeropathies do not acutely impair end protection and hematopoietic function in mice. (**A**) Schematic of human mutations and their equivalents in mice. (**B**) Experimental scheme, similar to Figure 4. (**C**) Survival of mice reconstituted with BM containing TPP1 rescue constructs noted in **B**. (**D**) EGFP expression in donor NUP98-HOXA10HD-mCherry^+^ CD11b^+^Gr-1^+^ myeloid cells before poly(I:C)-induced loss of endogenous TPP1 (d0) and subsequent time points. (**E** and **F**) EGFP expression in donor NUP98-HOXA10HD-mCherry^+^ LSK progenitor cells from BM at day 239 after poly(I:C) induction. *n* = 10 per group. ***P < 0.001 by log-rank Mantel-Cox test (**C**). *P < 0.05, ***P < 0.001 by 2-way ANOVA with post hoc Tukey’s test to assess differences in means (**D**). Mean and 1 SD reported.

Using a similar approach, we then turned our attention to human TPP1 mutants and variants that have been reported in patients with telomeropathies, including BM failure syndromes (47–49). We asked if mice harboring the TPP1 deletion mutation (ΔK82) equivalent to those patients suffering from telomeropathies also show acute hematopoietic defects. We generated retroviral constructs to express the following TPP1 mutants (Figure 7A): 1) a ΔK82 in-frame mutant lacking a critical lysine residue at the center of TPP1’s “TEL patch” previously reported to coordinate the TPP1-TERT interaction (equivalent to the human ΔK170 mutation); 2) a P365T TPP1 variant affecting the TIN2-binding domain (equivalent to human P491T, previously identified together with ΔK170 in an index patient with HH; and 3) a ΔK82/P365T TPP1 double mutant. Importantly, the ΔK170 mutation was previously shown to behave as a true separation-of-function mutant in cell culture by affecting end elongation but not end protection (47). Instead, the P491T mutation only showed a modest impact on TPP1-TIN2 binding in co-IP assays (Supplemental Figure 4A) (47), although the functional significance of these findings in vivo remains unclear.

To assess the impact of these variants on in vivo hematopoiesis, we cotransduced *Mx-Cre^+^ Acd^fl/–^* B6-CD45.2 HSCs with these constructs and NUP98-HOXA10HD-mCherry before reconstitution of lethally irradiated B6-CD45.1 mice, followed by poly(I:C) 6 weeks later (Figure 7B). While all recipients of MigR1-EGFP–transduced HSCs became moribund within 20 days after poly(I:C), consistent with hematopoietic failure, most recipients of HSCs transduced with WT TPP1 or with ΔK82, P365T, or ΔK82/P365T TPP1 mutants survived long-term after poly(I:C), consistent with rescue from acute hematopoietic failure (Figure 7C). Moreover, tracking blood CD11b^+^Gr1^+^ myeloid cells over time showed rapid selection for EGFP^+^ cells in all groups transduced with WT or mutant TPP1 constructs (Figure 7D). Delayed selection for EGFP^+^ cells was also apparent in blood B220^+^CD19^+^ B cells and TCRβ^+^ T cells, consistent with trilineage reconstitution driven by EGFP^+^ progenitors (Supplemental Figure 4, B and C). At the termination of the experiment 239 days after poly(I:C), we assessed EGFP expression among NUP98-HOXA10HD-mCherry^+^ BM hematopoietic stem and progenitor cells with a Lineage^–^Sca-1^hi^c-Kit^hi^ (LSK) phenotype (Figure 7, E and F). Close to 100% of BM LSK cells expressed EGFP in all surviving mouse groups, consistent with the capacity of WT TPP1 as well as ΔK82, P365T, and ΔK82/P365T TPP1 to sustain HSC persistence.

Taken together, these results indicate that TEL patch mutants identified in human patients are unlikely to impair TPP1’s end protection functions, consistent with a more selective impact on its end replication function (which does not become apparent within 1 generation in mice) (57). In addition, unlike Y376A/Y378A or R393A/L394A TPP1 mutants, the neighboring P365T variant in the TIN2-binding domain of TPP1 did not impair hematopoiesis, suggesting that it does not greatly affect the end protection functions of the shelterin complex and therefore the TPP1-TIN2 interaction in vivo.

## Discussion

### The TPP1-TIN2 interaction is more extensive than previously appreciated.

Using a homology-directed mutagenesis screen, we discovered that the TPP1-TIN2 interface is larger than previously appreciated. We found that TPP1 amino acids 496–507, as well as the previously characterized amino acids 510–544 of the TPP1_TBM_, are critical for facilitating the interaction between TPP1 and TIN2 in co-IP experiments. Therefore, we call this region the TPP1 TIN2-binding motif extension, or TPP1_TBM-ext_. The TPP1_TBM-ext_ is not only critical for TIN2 binding but also essential for bolstering the TIN2-TRF2 interaction.

The ability to detect TPP1-TIN2 binding defects seems dependent on the nature of the experimental approach, as the yeast-two hybrid domain-deletion analysis that led to the TPP1_TBM_ definition used in the crystal structure failed to reveal the TPP1_TBM-ext_ as a component of TIN2 binding. Along these same lines, a cell-based NAAIRS TPP1 mutagenesis screen using colocalization of mCherry-TIN2 with GFP-TPP1-LacI that coats a chromosomally integrated LacO array revealed that only a fraction of TPP1_TBM_ residues (aa 528–533) are critical for binding TIN2 (58). Disruption of other TPP1_TBM_ residues that were captured at the TPP1-TIN2 interface in the crystal structure or TPP1_TBM-ext_ residues that we report here did not alter TIN2 localization to the LacO-tethered TPP1 focus (58). We infer from these observations that co-IP provides an optimal platform for sensitive detection of shelterin protein-protein binding defects in a physiologically relevant context, in contrast to the LacO array system that may artificially increase local concentration to potentially mask binding defects. While our data are consistent with a newly identified interaction region between TPP1 and TIN2, future structural studies will be instrumental in understanding the full TPP1-TIN2 interface.

### Not all protein-protein interactions of TPP1 are made equal.

Our studies suggest that there is a strict hierarchy in the strength of the 3 protein-protein interactions that TPP1 participates in. The strongest interaction occurs with POT1, the telomeric ssDNA-binding protein that protects natural chromosomal ends from activating an ATR response. The difficulty of severing this interaction using single or double mutations in the interface combined with the low nanomolar/high picomolar affinity of the POT1-TPP1 complex for telomeric ssDNA (20) strongly suggests that the G-rich overhang at telomeres is constitutively protected by not just POT1, but rather the POT1-TPP1 heterodimeric complex. This conclusion is supported by our in vivo findings using hematopoietic homeostasis as a sensitive readout for end protection, as a targeted TPP1 mutation affecting the POT1-TPP1 interaction completely disrupted the capacity of TPP1 to rescue HSC function.

Depictions of shelterin in this and previous studies show a 6-membered complex, suggesting that the TPP1-TIN2 interaction is constitutive (Figure 1A). Our analysis of TPP1_TBM-ext_ mutations in vivo confirms the importance of this interaction in mammalian cells, as a double mutant targeted to the TPP1_TBM-ext_ region failed to rescue hematopoiesis or the TIF response in cells lacking endogenous TPP1. However, the ease with which single/double mutations disrupt the TIN2-TPP1 interface suggests the possibility that this interaction is physiologically reversible. Indeed, several phosphorylation sites have been identified on both TPP1 and TIN2 (59–61), although it is not clear if or how they affect interactions of these proteins within shelterin.

The TPP1-TERT interaction is unique among the 3 interactions of TPP1 in that it occurs transiently during DNA replication in a narrow window of S-phase of the cell cycle (62). Previous studies have implicated both POT1 and TIN2 in enhancing the telomerase-related activities of TPP1. For example, POT1-TPP1, but not TPP1 alone, increases the enzymatic repeat addition processivity (ability of telomerase to add multiple telomeric repeats in 1 primer binding event), likely through tethering of telomerase-TPP1 to POT1-bound DNA (20). POT1 also increases the association of TPP1 with telomerase in co-IP experiments (45). Along similar lines, a recent study suggested that the presence of TIN2 increases the activity of POT1-TPP1–associated telomerase (58, 63). This suggests either a direct interaction between TIN2 and telomerase or an indirect role for TIN2 in enabling allosteric/stability changes in the POT1-TPP1 complex that improve telomerase association. While there is no evidence for a direct TIN2-telomerase interaction, it must be noted that a cluster of TIN2 residues with no known binding partner represents a major mutational hotspot in telomeropathies (59, 63–67). While interactions with shelterin binding partners enhance the functionality of the telomerase-TPP1 interaction, they do not explain how telomerase recruitment to telomeres is switched off outside S-phase. The switch for telomerase-TPP1 binding could be provided by posttranslational modification of TPP1/TERT or by binding of this complex to factors outside of shelterin, although evidence for either of these scenarios is currently lacking.

### Role of the TPP1 binding surfaces in normal physiology and disease.

Our findings contribute to a refined molecular understanding of TPP1’s functions at the core of the shelterin complex. In hematopoiesis, both global loss of TPP1 and selective disruption of its interfaces with POT1 and TIN2 led to rapid hematopoietic failure, HSC loss, and evidence of cell cycle arrest within days after inactivation of endogenous *Acd* in mice. We previously reported findings of an unleashed DNA damage response as well as evidence of chromosomal instability and end-end fusions after loss of TPP1 in hematopoietic stem and progenitor cells, consistent with chromosomal end deprotection being the dominant early impact in this cellular context (31). TPP1 loss triggered a p53-dependent response, but p53 inactivation was insufficient to rescue HSC survival, indicating more widespread effects. In our current study, carefully selected TPP1 mutations that disrupt TPP1’s interaction with TIN2 or POT1 were completely ineffective at rescuing the end protection functions of endogenous TPP1 in mice. Thus, TIN2-TPP1-POT1 interactions underlie a central mechanism of shelterin function whose disruption has much more rapid consequences than the progressive loss of telomere length observed upon disruption of end replication.

In contrast, we modeled the effects of a patient mutation found in DC-related disorders that specifically affects TPP1’s TEL patch, which recruits telomerase to telomeres. This mutant had no detectable acute effect on hematopoiesis in mice, even in a system that is highly sensitive to any perturbations in end protection. These findings are consistent with a selective impact on end replication, which does not lead to acute effects in mouse hematopoiesis due to the extensive time needed to achieve critical shortening of long mouse telomeres. Thus, the ΔK170 TEL patch TPP1 mutant behaves as a pure separation-of-function mutant in vivo. One of the original families harboring the ΔK170 mutation included a proband with a compound heterozygous mutation associating ΔK170 with a P491T amino acid change on the second *ACD* allele. This change maps to the TIN2-binding domain, a region that we show is critical for end protection in hematopoiesis. While the TPP1 P491T mutation resulted in a moderate loss of TIN2 binding in vitro (47), expression of P491T alone or in combination with ΔK170 did not induce functional hematopoietic defects in our system, even when endogenous *Acd* was inactivated. Thus, the clinical significance, if any, of this P491T cannot be attributed to defects in end protection. Moving forward, our careful molecular mapping of TPP1’s molecular interfaces could function as a blueprint to understand and predict the consequences of human mutations, and this approach could be extended to other elements of the shelterin complex, such as TIN2 and POT1, that demonstrate recurrent mutations in human disease.

## Methods

### Molecular cloning and site-directed mutagenesis.

All TPP1 mutations were introduced into the p3x-FLAG-TPP1-cDNA6/myc-HisC vector using QuikChange Site-Directed Mutagenesis Kit (Agilent Technologies) and complementary mutagenic primers (Integrated DNA Technologies). The resulting FLAG-TPP1 plasmids were sequenced to confirm the presence of the intended mutation and the absence of errors that may have been introduced during PCR amplification. The 3x-FLAG–tagged TRF2 and 6x-Myc–tagged POT1 and TIN2 for human cell expression were cloned into the pTET-IRES-eGFP-Bi4 vector for use in co-IP experiments. Additionally, 6x-Myc–tagged TIN2 and 3x-FLAG–tagged TIN2 and TRF2 were cloned into a pcDNA3-derived vector. This pcDNA3-derived, 3x-FLAG–tagged TIN2 vector was then used for subsequent site-directed mutagenesis exactly as described above to generate FLAG-TIN2_A15R_, FLAG-TIN2_F152A_, FLAG-TIN2_E153A_, FLAG-TIN2_K160A_, and FLAG-TIN2_W198A_.

### Co-IP.

Co-IP experiments were performed exactly as described previously (46). Briefly, HeLa-EM2-11ht cells (68) were transfected with 1 μg of each plasmid. Twenty-four to 48 hours after transfection, cells were trypsinized, resuspended in medium containing 50% FBS, and pelleted. Cells were then resuspended in 400 μL of lysis buffer, 50 mM Tris-HCl (pH 7.6), 20% glycerol, 1 mM EDTA, 150 mM NaCl, 0.5% Triton X-100, 0.02% SDS, 1 mM dithiothreitol, 2 mM phenylmethylsulfonyl fluoride, and complete protease inhibitor cocktail (Roche) and kept on ice. Then 33 μL of 4 M NaCl and 433 μL of water were added, and lysates were spun down using centrifugation (16,000*g*, 10 minutes at 4°C). Then 40 μL of supernatant was added to SDS gel loading buffer and kept aside for analysis of input samples. The remaining lysate was used directly for immunoprecipitation. For FLAG immunoprecipitation, lysate was added to 30 μL of prewashed anti-FLAG M2 affinity gel (MilliporeSigma; A2220) and incubated overnight at 4°C. For Myc immunoprecipitation, 5 μL of c-Myc antibody (DSHB; 9E 10) was added, and lysates were incubated for 2–4 hours at 4°C. After antibody incubation, lysate was transferred to tubes containing 30 μL of prewashed protein A/G agarose (Pierce, Thermo Fisher Scientific; 20421) and incubated overnight at 4°C. After overnight incubation, beads were washed, and protein was eluted from the beads by adding 60 μL of 2× SDS gel loading buffer. All samples were analyzed by SDS-PAGE followed by immunoblotting with HRP-conjugated anti-FLAG or anti-Myc antibodies.

### Immunoblotting.

Immunoblotting was performed using standard procedures and exactly as described previously (46). The following antibodies were used for detection with chemiluminescence by ECL plus reagents (Pierce ECL Western Blotting Substrate; Thermo Fisher Scientific): mouse monoclonal anti-FLAG M2-HRP conjugate (MilliporeSigma; A8592; 1:10,000), mouse monoclonal anti–c-Myc (9E10) HRP conjugate (Santa Cruz Biotechnology; sc-40 HRP; 1:10,000), rabbit polyclonal anti-TPP1 antibody (Bethyl Laboratories, A303-069A, 1:500), and anti-rabbit HRP-conjugated secondary antibody (Jackson ImmunoResearch; 111035045). The data were visualized using a gel documentation system (ChemiDoc MP System; Bio-Rad). See complete unedited blots in the supplemental material.

### HeLa cell culture.

HeLa-EM2-11ht cells (68) were cultured exactly as described previously (46) at 37°C in the presence of 5% CO_2_ and propagated in modified DMEM (Gibco, Thermo Fisher Scientific, 11995-065) containing 100 U/mL penicillin, 100 μg/mL streptomycin, and 10% FBS.

### MEF culture and transduction with lentiviruses.

*Acd^fl/fl^* MEFs immortalized with SV-40 (gift from Titia de Lange, Rockefeller University, New York, New York, USA) (56) were cultured in DMEM containing 15% heat-inactivated FBS (Corning), 100 μg/mL streptomycin, and 100 U/mL penicillin. For generating Cre-ER lentivirus, 2 μg of MSCV-puro-CreER (gift from Andrew Muntean, University of Michigan), along with 2 μg each of packaging vectors pCgp-MoMULV-gag-pol and pHIT123-MLV-ecotropic env-SV40 ori, were transfected into HEK293T cells (ATCC) at 60% confluence in a 6-well format using Lipofectamine LTX (Invitrogen, Thermo Fisher Scientific). The supernatant containing virus particles was collected after 24 hours and 48 hours, pooled, and concentrated using the LentiX concentrator (Takara Bio). The concentrated viral particles were added to 50% confluent MEFs in a 6-well format, along with polybrene (8 μg/mL; MilliporeSigma). The medium was replaced with regular growth medium the next day. After another 24 hours, selection with 2 μg/mL puromycin, along with a kill control well, was started. Lentiviruses for mTPP1 WT and variants were prepared using the same packaging vectors. Concentrated viruses were used to transduce the newly established Cre-ER MEFs *Acd^fl/fl^* line at 60% confluence in a 6-well format. After 20 hours, the cells were split onto coverslips. After 6 hours, the medium was replaced with medium containing 0.5 μM 4-OHT (MilliporeSigma). This was considered time point 0. At the 72-hour time point, coverslips were fixed for subsequent TIF analysis.

### TIF analysis.

Telomere FISH was performed before immunofluorescence for 53BP1. Coverslips containing MEFs were washed twice in PBS. Cells were fixed in 4% formaldehyde in PBS for 10 minutes at room temperature (RT). Cells were permeabilized in 0.5% Triton X-100 in PBS for 10 minutes at RT and washed twice in PBS. Cells were rehydrated in 50% formamide–2X SSC for 5 minutes. Coverslips with cells facing down were placed on hybridization solution supplemented with 0.05 μM (0.3 μg/mL) Cy3-labeled PNA-(CCCTAACCCTAACCCTAA) telomere probe. Hybridization solution contained 100 mg/mL dextran sulfate, 0.125 mg/mL *E*. *coli* tRNA, 1 mg/mL nuclease-free BSA, 0.5 mg/mL salmon sperm DNA, 1 mM vanadyl ribonucleoside complexes, and 50% formamide in 2X SSC. The coverslips were hybridized at 80°C on a heat block for 6 minutes and incubated in the dark for 2 hours. They were washed twice with 50% formamide–2X SSC, washed twice with PBS, fixed again in 4% formaldehyde PBS for 10 minutes, and washed again twice in PBS before being processed for immunofluorescence. Coverslips were blocked in blocking buffer (1 mg/mL BSA, 3% goat serum, 0.1% Triton X-100, 1 mM EDTA, pH 8) for 30 minutes, incubated with anti-53BP1 rabbit primary antibody (Novus Biologicals; NB100-304; 1:1000) for 2 hours at RT, washed 3 times in PBS, incubated with goat anti-rabbit secondary antibody conjugated to Alexa Fluor 647 (Life Technologies, Thermo Fisher Scientific; A21244; 1:500) for 30 minutes at RT, washed 3 times in PBS, mounted on microscope slides with ProLong Gold antifade reagent with DAPI (Life Technologies, Thermo Fisher Scientific), and sealed with clear polish.

### Telomere localization analysis.

Telomere localization of 3X FLAG-tagged TPP1 and TIN2 constructs was performed as described previously for FLAG-tagged TPP1 variants and isoforms (46, 51).

### Microscopy.

A laser-scanning confocal microscope (SP5; Leica Microsystems) equipped with a 100× oil objective was used to image immunofluorescence and FISH experiments. The images were processed with ImageJ (NIH) and Adobe Photoshop, and colocalizations were quantified manually. A total of 51 cells were counted for each condition.

### Mice.

C57BL/6(B6)-CD45.2 or C57BL/6(B6)-CD45.1 mice (The Jackson Laboratory) were used for all transplantation studies. *Acd*-floxed conditional (*Acd^fl^*) and null alleles (*Acd*^–^) were crossed with *Mx1-Cre*, as described (31). Activation of *Mx1-Cre* expression was achieved with 5 i.p. injections of 200 μg poly(I:C) every other day (GE Healthcare). Mice were bred and studied per protocols approved by the University of Michigan’s IACUC and the University of Pennsylvania’s Office of Regulatory Affairs.

### BM retroviral transduction and transplantation.

Production of MSCV-based retroviral vectors was performed in HEK293T cells, followed by semiquantitative titration on 3T3 cells (ATCC), as described (69). On day –4, *Mx-Cre^+^ Acd^fl/–^* donor B6-CD45.2 mice were injected i.p. with 150 mg/kg 5-FU to induce HSC cycling, as described (70). On day 0, BM cell suspensions were obtained from femurs and tibia, pooled, and counted with a hemocytometer. Cells were plated at 5 × 10^5^ cells/mL in culture media of DMEM, pen/strep, glutamine, 15% FBS, IL-3 (6 ng/mL), IL-6 (10 ng/mL), and stem cell factor (100 ng/mL) (PeproTech). On day 2, cells were collected and cotransduced with an MSCV-based retrovirus expressing NUP98-HOXA10HD (53–55) and mCherry downstream of an internal ribosomal entry site (IRES), and an MSCV-based retroviral vector expressing TPP1 WT, L191A/L193A, Y376A/Y378A, R393A/L394A, ΔK82, P365T or ΔK82/P365T, and IRES-EGFP versus IRES-EGFP alone (MigR1). Transduction was performed in culture media plus polybrene (4 μg/mL). On day 4, cells were collected and replated in fresh culture media. On day 7, cells were collected for expansion, analyzed by flow cytometry for mCherry and EGFP expression, and replated at 10% of the original density. On day 10, cells were collected, analyzed by flow cytometry, and injected i.v. into lethally irradiated recipient mice (11 Gy split in 2 fractions, Cesium-137 source).

### Complete blood counts.

Peripheral blood counts were acquired on a Sysmex XT-2000iV Automated Hematology Analyzer within 4 hours of collection in EDTA tubes.

### Antibodies and flow cytometry.

Flow cytometry was performed on peripheral blood or BM single-cell suspensions after red blood cell lysis. All antibodies were from BioLegend: CD11b (clone M1/70), Gr1 (clone RB6-8C5), CD19 (clone 6D5), B220 (clone RA3-6B2), TCRβ (clone H57-597), c-Kit (clone 2B8), Sca-1 (clone D7), CD45.1 (clone A20), CD45.2 (clone 103). To assess progenitor population, mature cells were excluded by a lineage cocktail including anti-CD11b, –Gr-1, -B220, -CD19, -TCRβ, -CD8α (clone 53-6.7), -CD3 (clone 17A2), -TCRγ/δ (clone GL3), -CD11c (clone N418), -NK1.1 (clone PK136), and -Ter119 (all from BioLegend). Nonviable cells were excluded from analysis with Zombie Aqua Fixable Viability Dye (BioLegend), 7-amino-actinomycin D (BioLegend), or DAPI (MilliporeSigma). Flow cytometric analysis was performed using a 4-laser Fortessa (BD). FlowJo (Tree Star) was used for data analysis.

### Statistics.

All statistical tests were performed using Prism software (GraphPad version 8). Unless otherwise noted, experiments with more than 2 groups were analyzed for differences with 1-way ANOVA or 2-way ANOVA depending on the number of factors. If a factor significantly explained the variation of the data, multiple comparisons between groups were made with post hoc Tukey’s tests adjusted for multiple comparisons, assuming α = 0.05. The multiple comparisons performed are represented in the figures by lines between groups. Survival curves were compared using a log-rank (Mantel-Cox) test. Graphs were generated in GraphPad Prism and presented as mean ± SD. *P* < 0.05 was considered significant.

### Study approval.

All mouse studies were conducted according to approved protocols by the University of Pennsylvania’s Office of Regulatory Affairs or the University of Michigan’s IACUC.

## Author contributions

SG, JN, IM, and CEK conceived the study. SG conducted the in vitro mutagenesis screening experiments with help from SP on co-IP experiments. SP performed TIF analysis. AF, EP, and FA conducted the hematopoiesis experiments under the supervision of IM and RK. VMT generated sequence alignments and analyzed crystal structures to help design TIN2 mutants. JC performed pilot hematopoiesis experiments. All authors contributed to data analysis, and SG, SP, JN, EP, AF, and IM wrote the manuscript with input from all authors.

## Supplementary Material

Supplemental data

## Version 1 04/06/2021

In-Press Preview

## Version 2 05/10/2021

Electronic publication
