# Supplementary material for: TPP1 mutagenesis screens unravel shelterin interfaces and functions in hematopoiesis
Source: JCI Insight. 2021 May 10;6(9):e138059. doi: 10.1172/jci.insight.138059 (PMC8262337; doi:10.1172/jci.insight.138059)

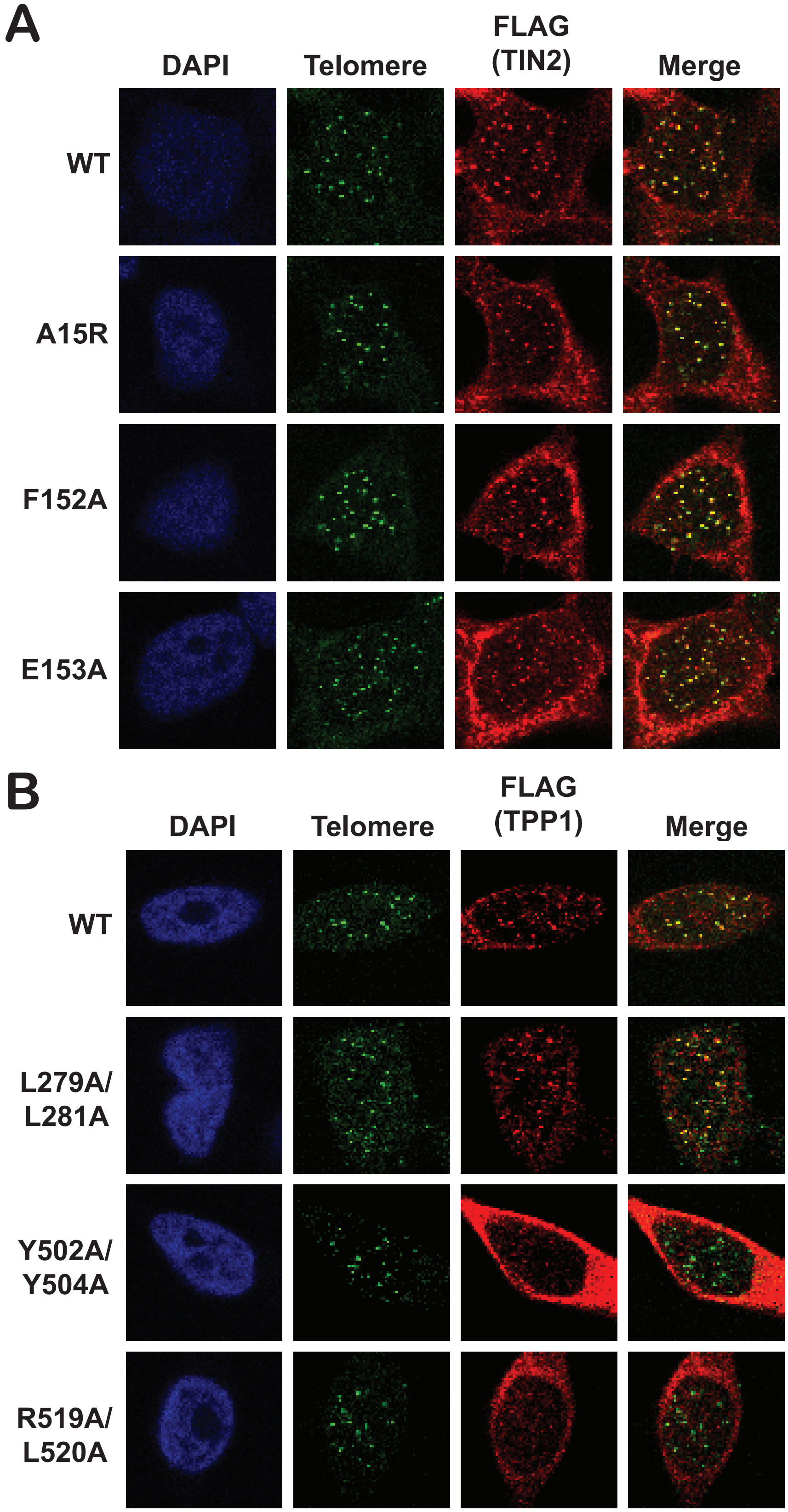

Supplemental Figure 1 related to Fig. 2 and Fig. 3. Telomere localization of TPP1 and TIN2 mutants. IF-FISH was used to analyze localization of indicated TIN2 (panel A) and TPP1 (panel B) WT and mutant constructs at telomeres. "FLAG (TIN2)" and "FLAG (TPP1)" show the FLAG immunofluorescence signal (red), and "Telomere" indicates FISH signal for telomeric DNA probed with a Cy3-labeled PNA (green). Yellow/Orange foci in the "Merge" panel implies colocalization of TIN2/TPP1 variants with telomeric DNA.

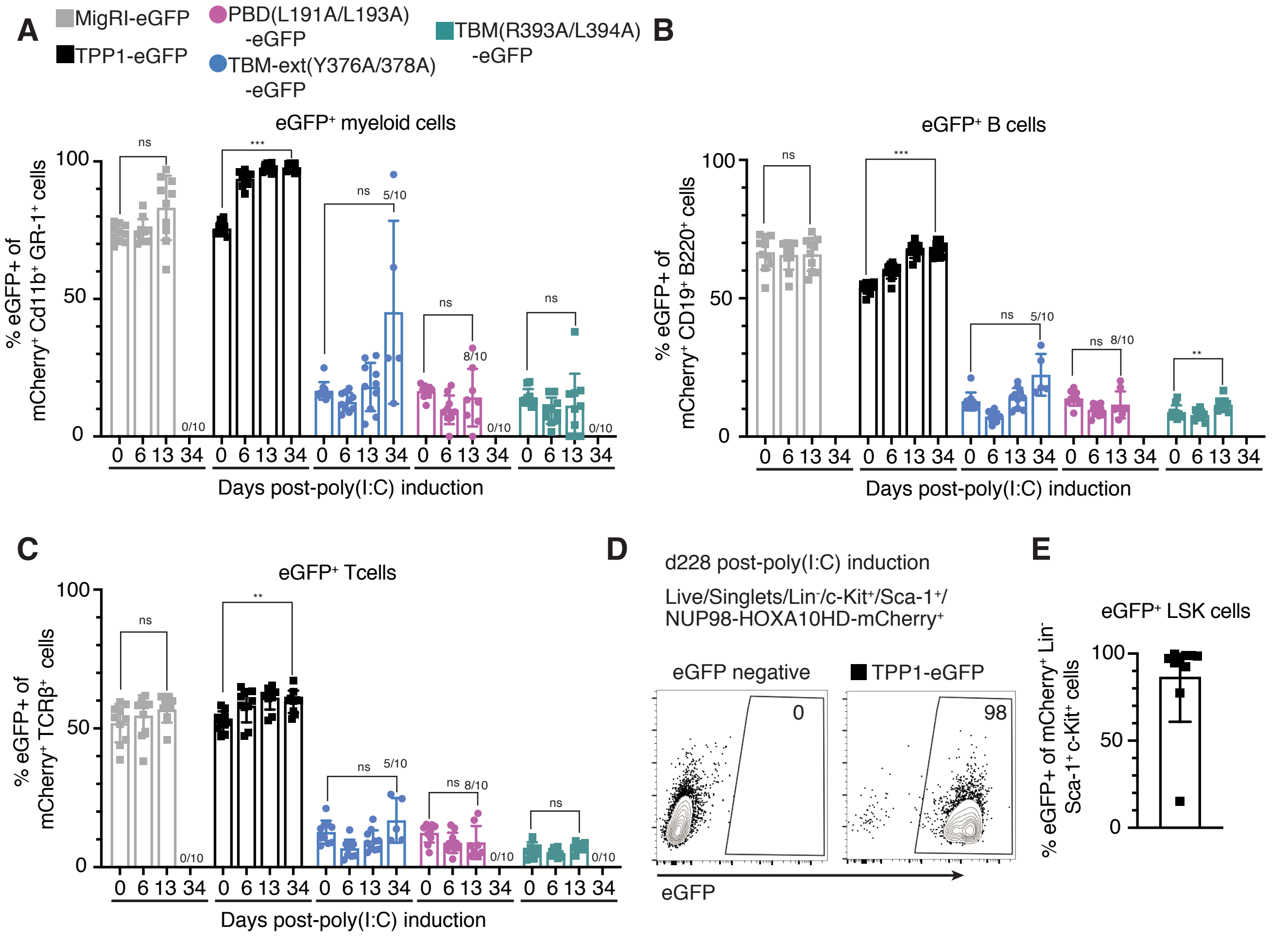

Supplemental Figure 2 related to Fig 5. Mutations in the TPP1$_{PBD}$, TPP1$_{TBM\text{-}ext}$, or TPP1$_{TBM}$ impair tri-lineage hematopoietic reconstitution. Donor NUP98-HOXA10HD-mCherry^{+} CD11b^{+}Gr-1^{+} myeloid cells **(A),** CD19^{+} B220^{+} B cells **(B),** and TCR $^{+}$ T cells **(C)** were assessed for expression of TPP1 rescue constructs by flow cytometric analysis of eGFP reporter before poly(I:C)-induced loss of endogenous *Acd* (d0) and at noted timepoints thereafter. n=10 per group, remaining numbers of mice are noted in the figure. **(D)** eGFP expression in donor Nup98AHoxA10mCherry^{+} Lineage^{-} c-kit^{+} Sca-1^{+} progenitor cells from bone marrow at d228 after poly(I:C) induction, summarized in **(E)**. **p <0.01 ***p <0.001 by two-way ANOVA with post hoc Tukey tests to assess differences in means. Mean and one SD reported.

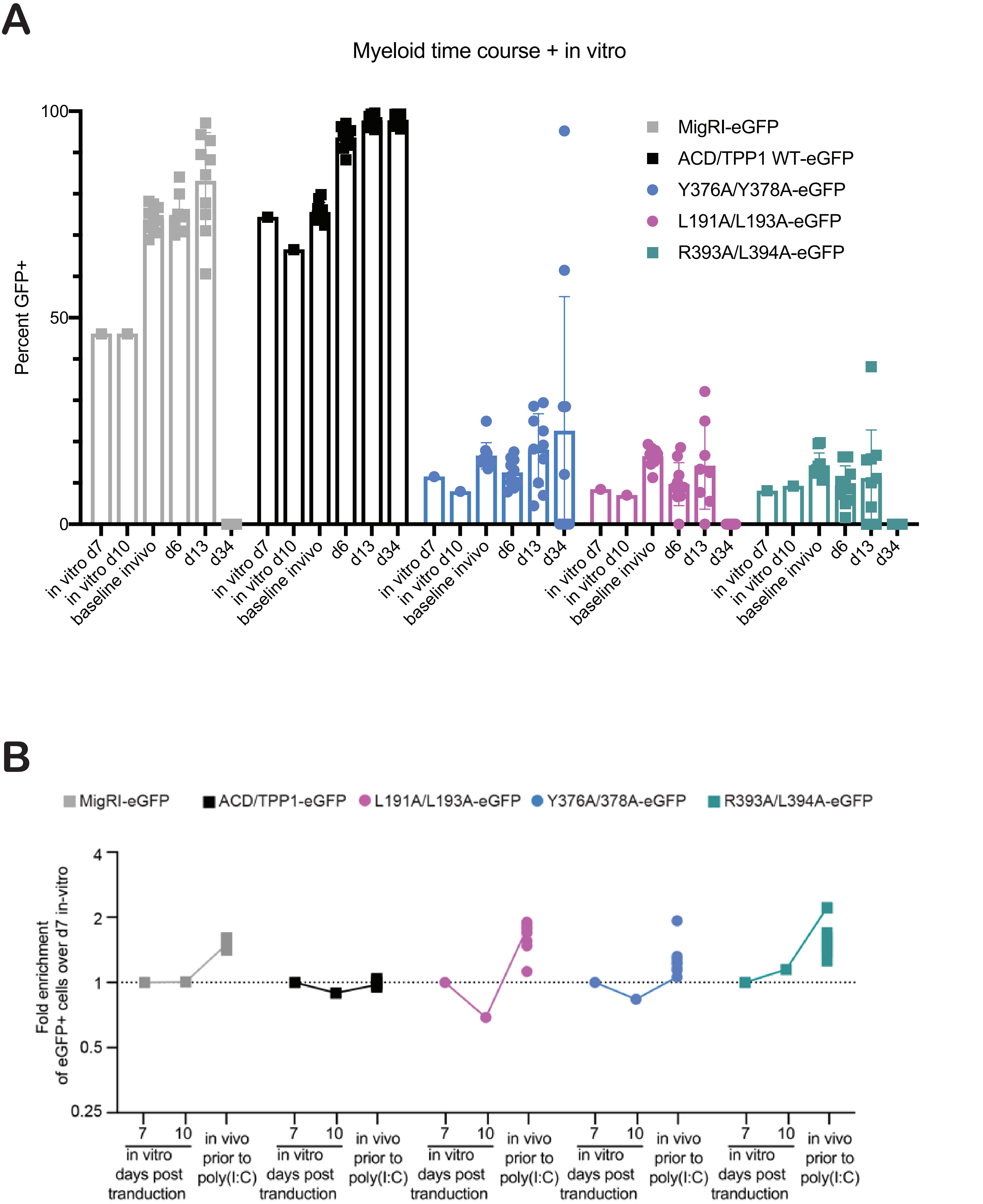

Supplemental Figure 3 related to Fig. 5: Before loss of endogenous Acd, stable ex vivo expansion and in vivo engraftment of NUP98-HOXA10HD-mCherry+ Mx-Cre^{+} Acd$^{fl/-}$ B6-CD45.2 hematopoietic progenitors expressing eGFP alone vs. eGFP and wild-type TPP1 vs. eGFP and the following TPP1 mutants: TPP1PBD (L191A/L193A), TPP1TBM (Y376A/Y378A), or TPP1TBM-ext (R393A/L394A). (A) Percentage of eGFP+ cells among mCherry+ cells at day 7 (d7) and day 10 (d10) of ex vivo culture and transduction, as well as percentage of eGFP+ cells among mCherry+ blood CD11b+Gr1+ myeloid cells at 6 weeks after transplantation (baseline in vivo) and at d6, d13 and d34 after poly(I:C) administration to inactivate endogenous Acd. Note that d6, d13 and d34 data are also shown in Fig. S2A, but repeated here for comparison with in vitro time points. (B) Relative expression of eGFP among mCherry+ cells at d7 and d10 of transduction and ex vivo expansion, as well as before poly(I:C) in vivo (normalized to 1 at day 7 post-transduction).

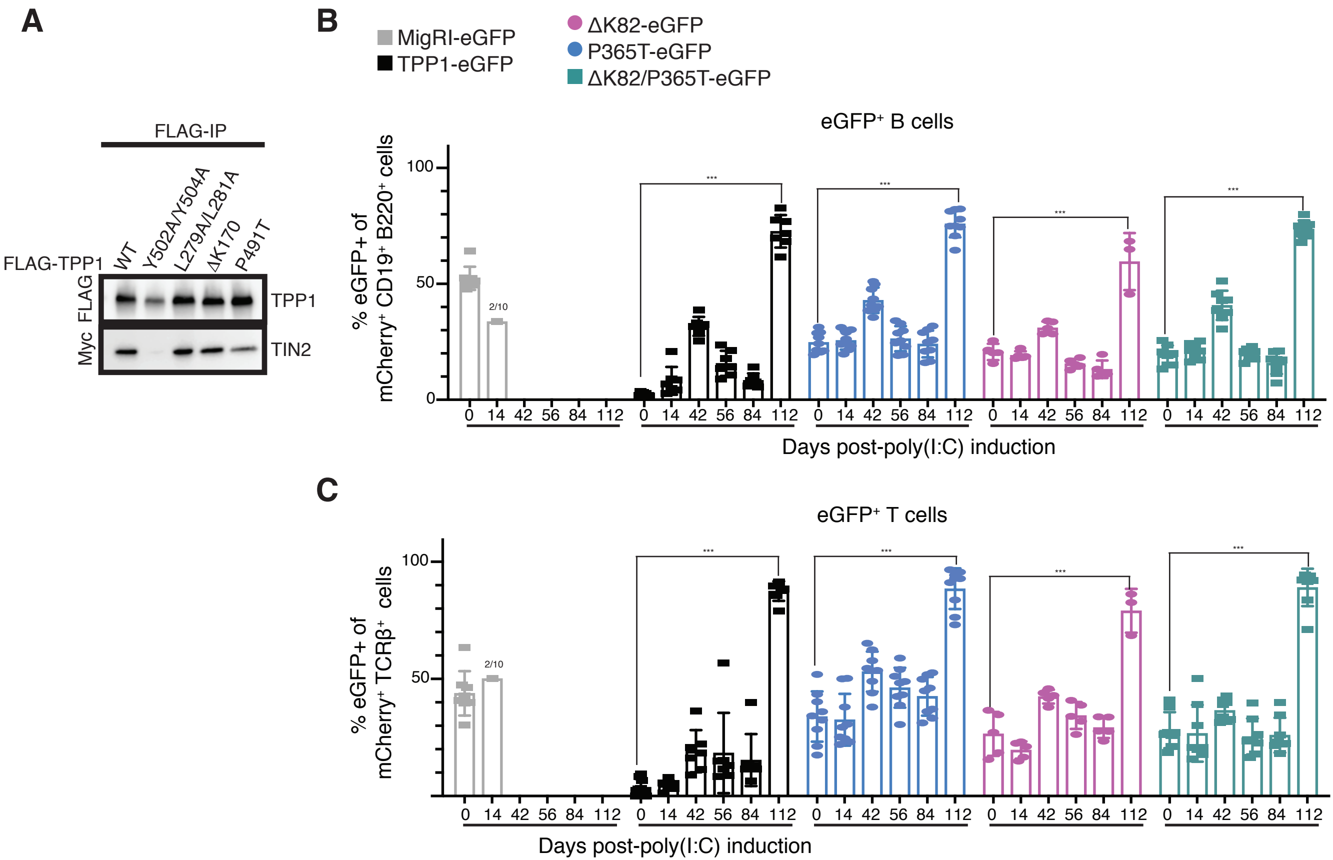

Supplemental Figure 4 related to Fig 7. TPP1 mutants from human patients with telomeropathies do not acutely impair tri-lineage hematopoiesis in mice. (A) Pulldown of indicated, transiently expressed FLAG-TPP1 mutants on anti-FLAG conjugated beads with Myc-TIN2. Donor NUP98-HOXA10HD-mCherry+ CD19+B220+ B cells (B), and TCRβ+ T cells (C) were assessed for expression of TPP1 rescue constructs by flow cytometric analysis of eGFP reporter before poly(I:C)-induced loss of endogenous Acd (d0) and at noted time points thereafter. n=10 per group, remaining numbers of mice are noted in the figures. ***p <0.001 by two-way ANOVA with post hoc Tukey tests to assess differences in means. Mean and one SD reported in figures.

Full unedited blots for Figure 2A

Left: Middle: Right:

Top (FLAG)

Bottom (Myc)

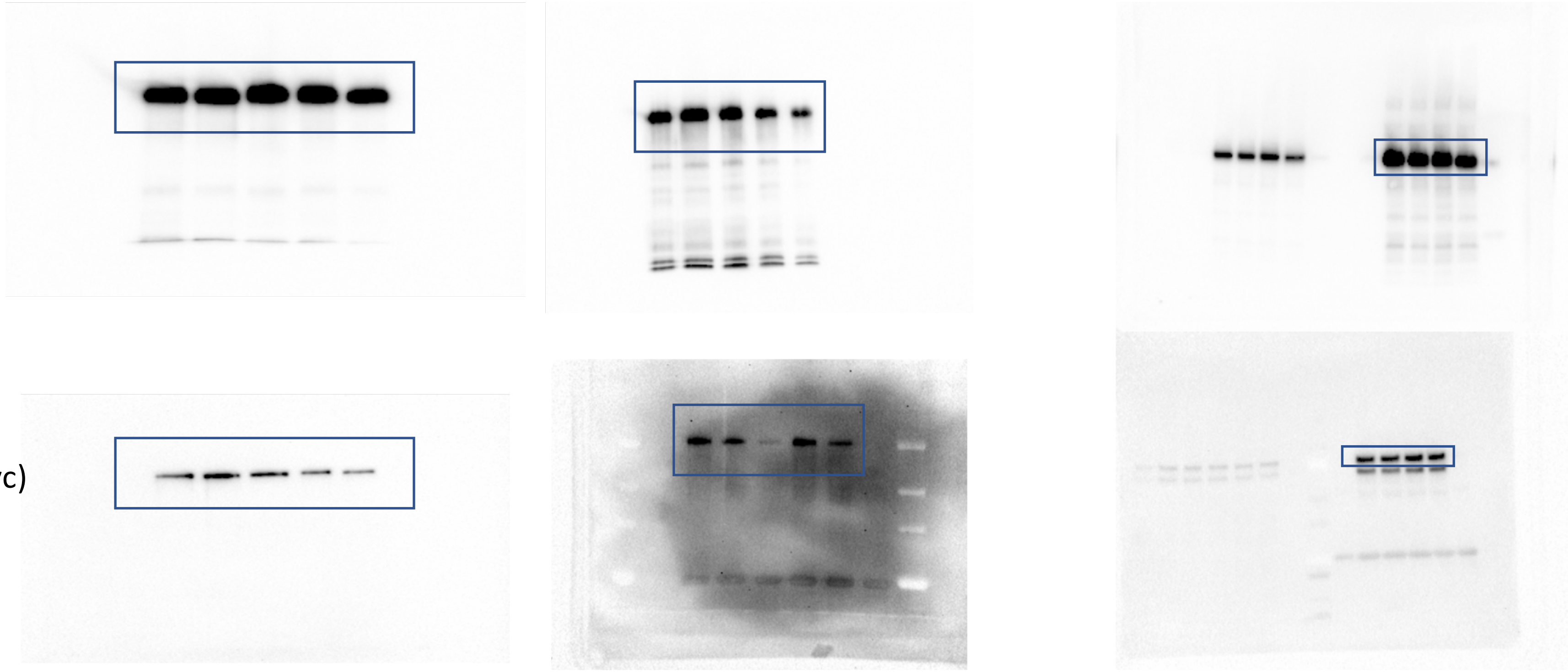

Full unedited blots for Figure 2B

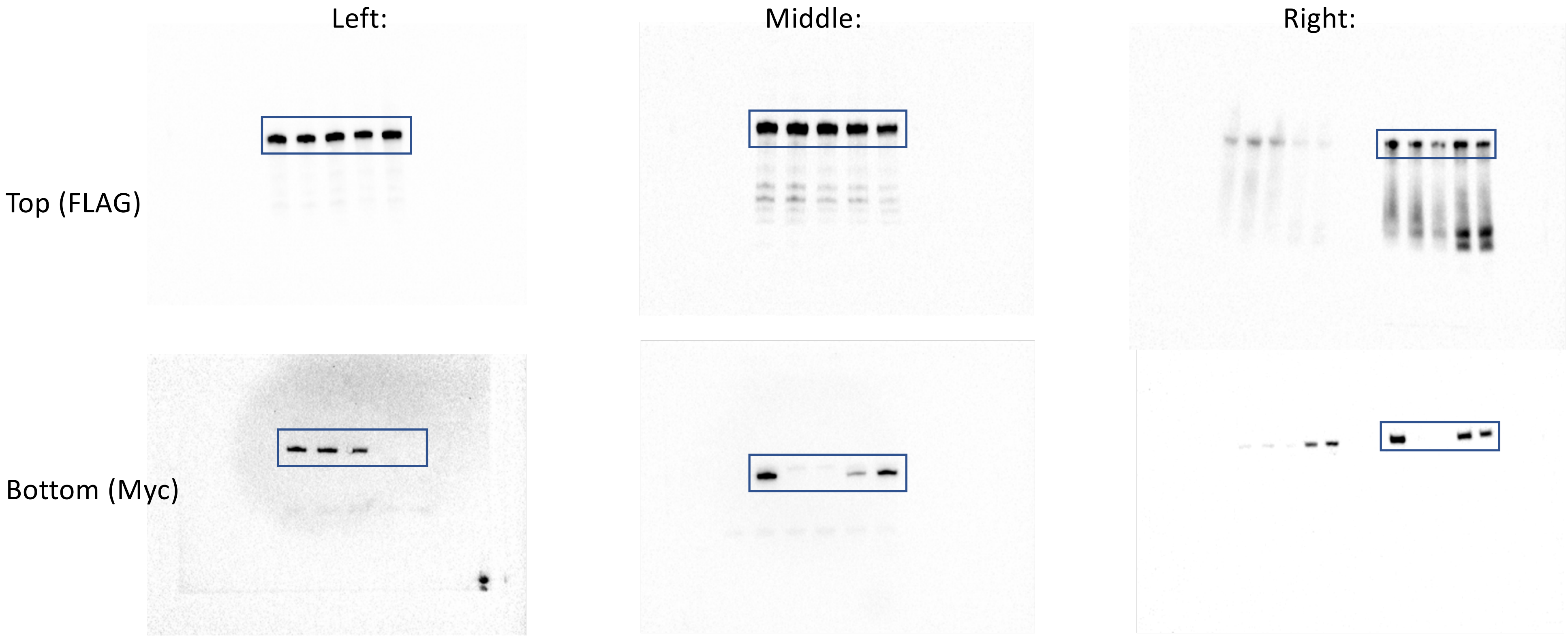

Full unedited blots for Figure 3A

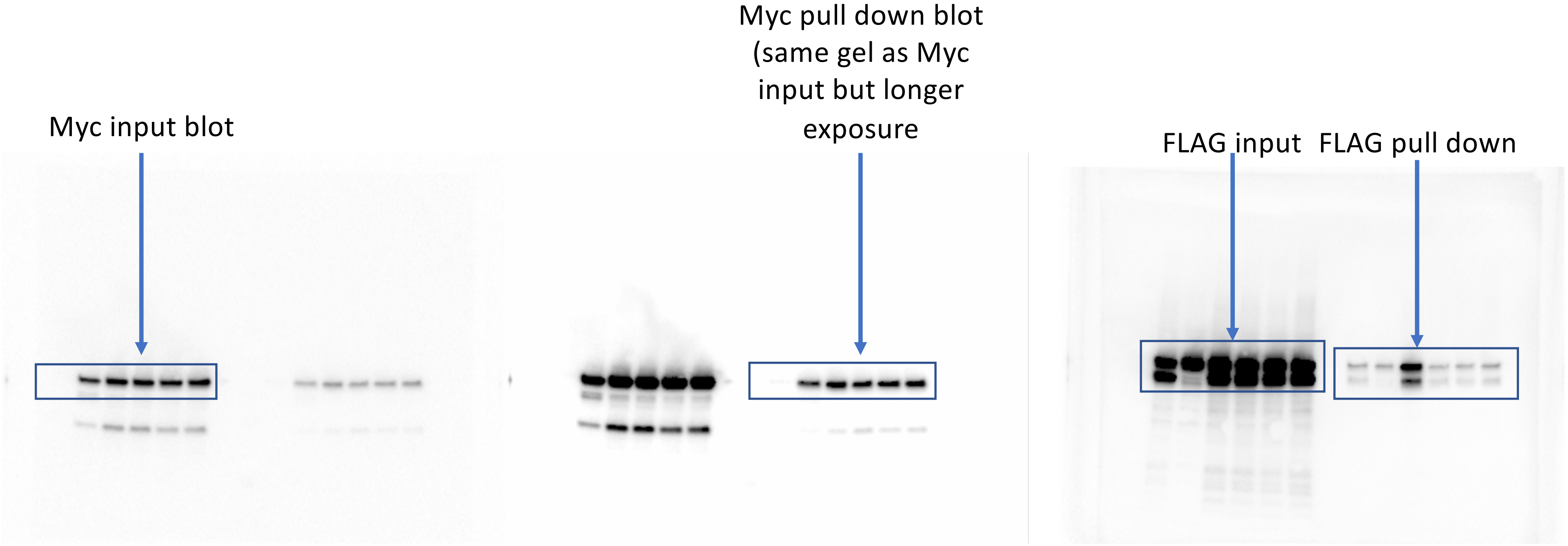

Full unedited blots for Figure 3C

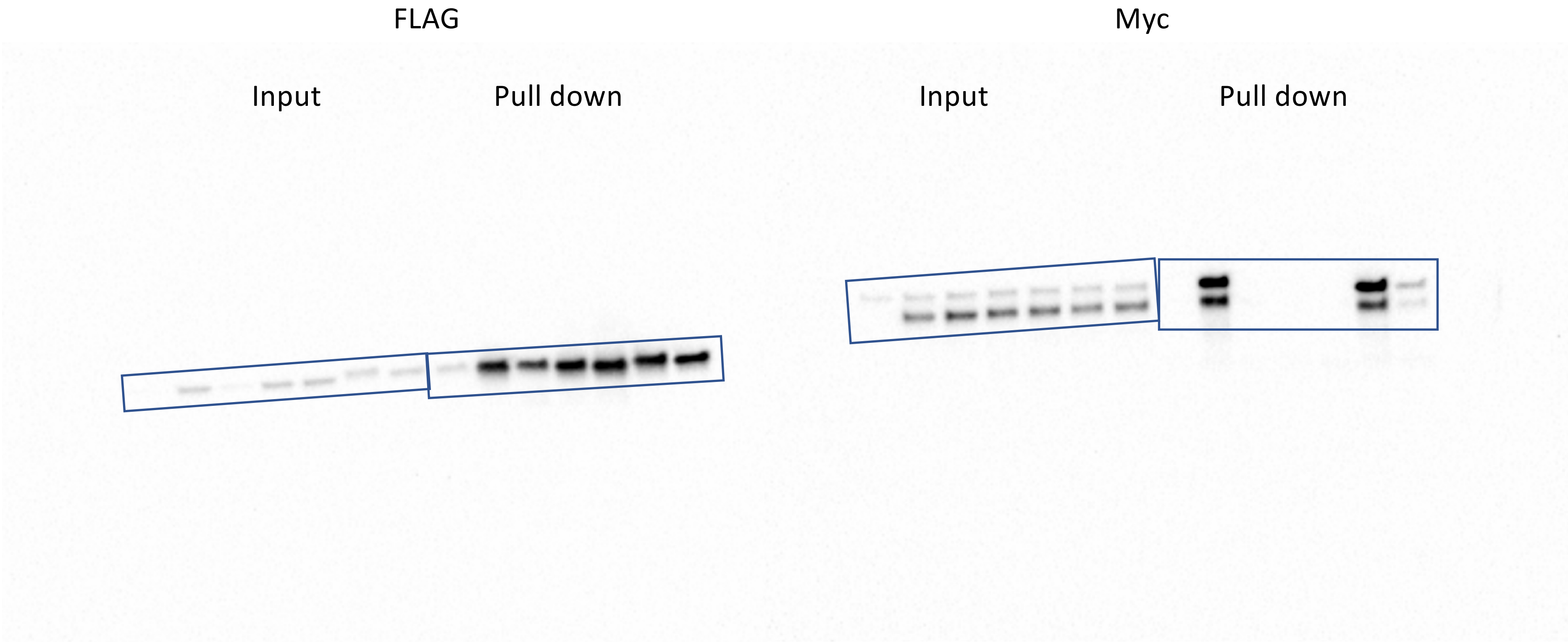

Full unedited blots for Figure 6A

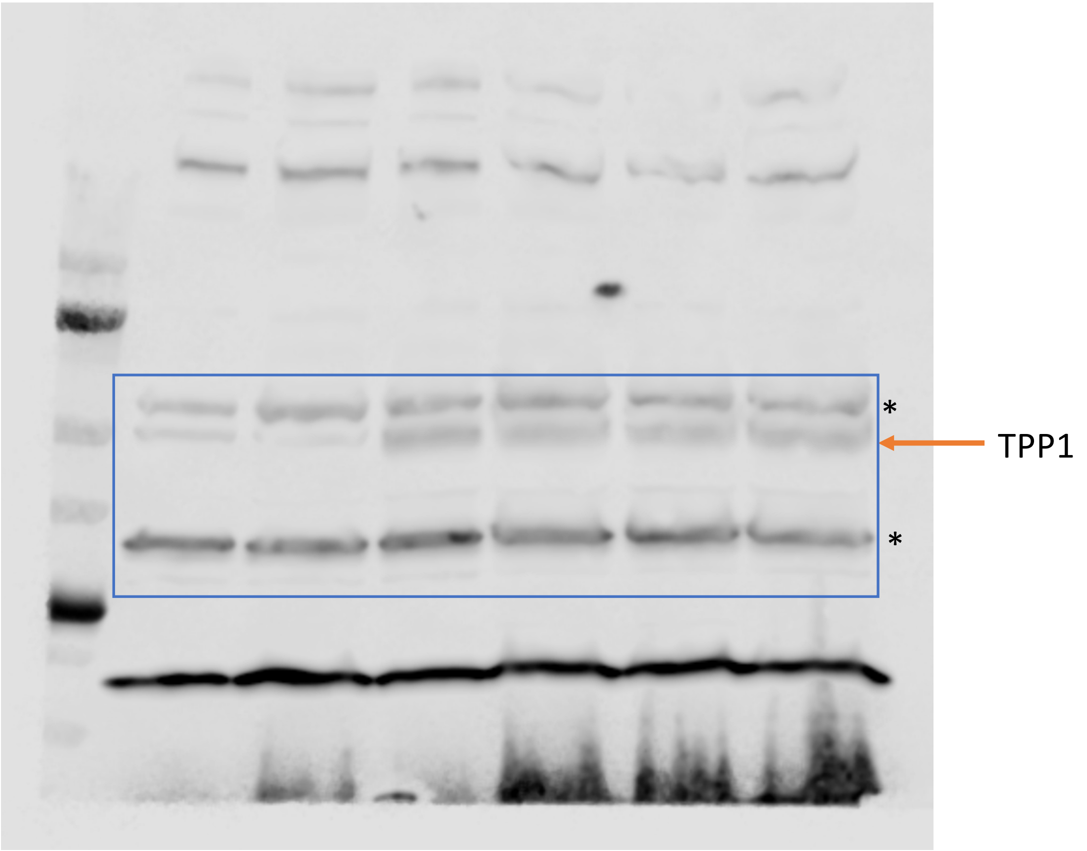

Full unedited blots for Figure S4A

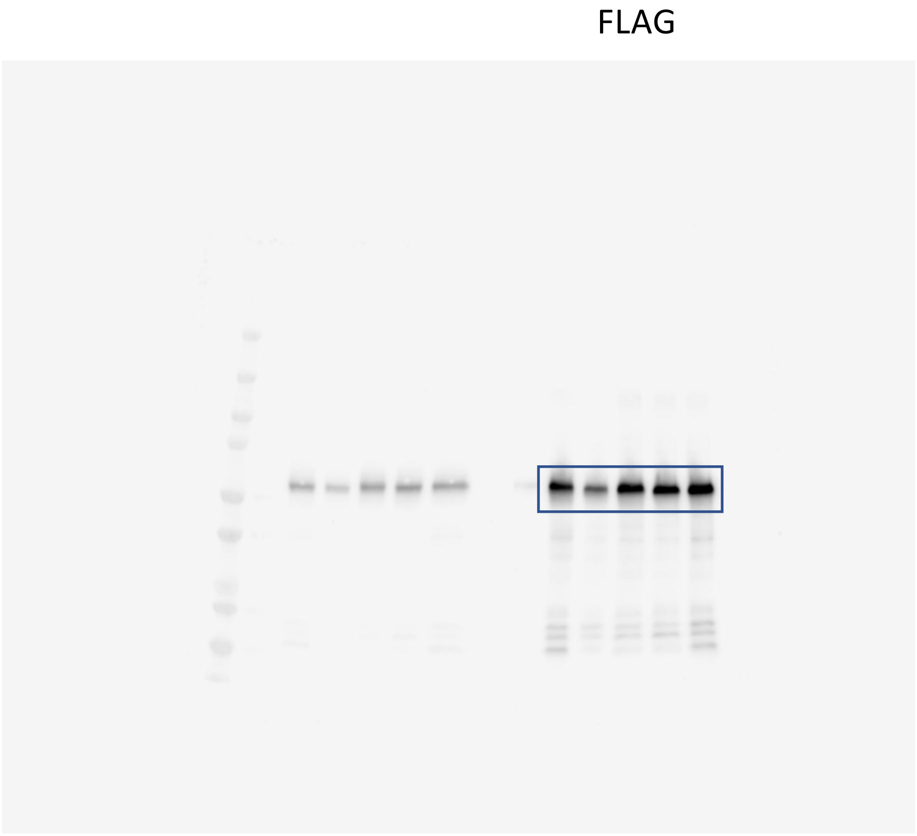

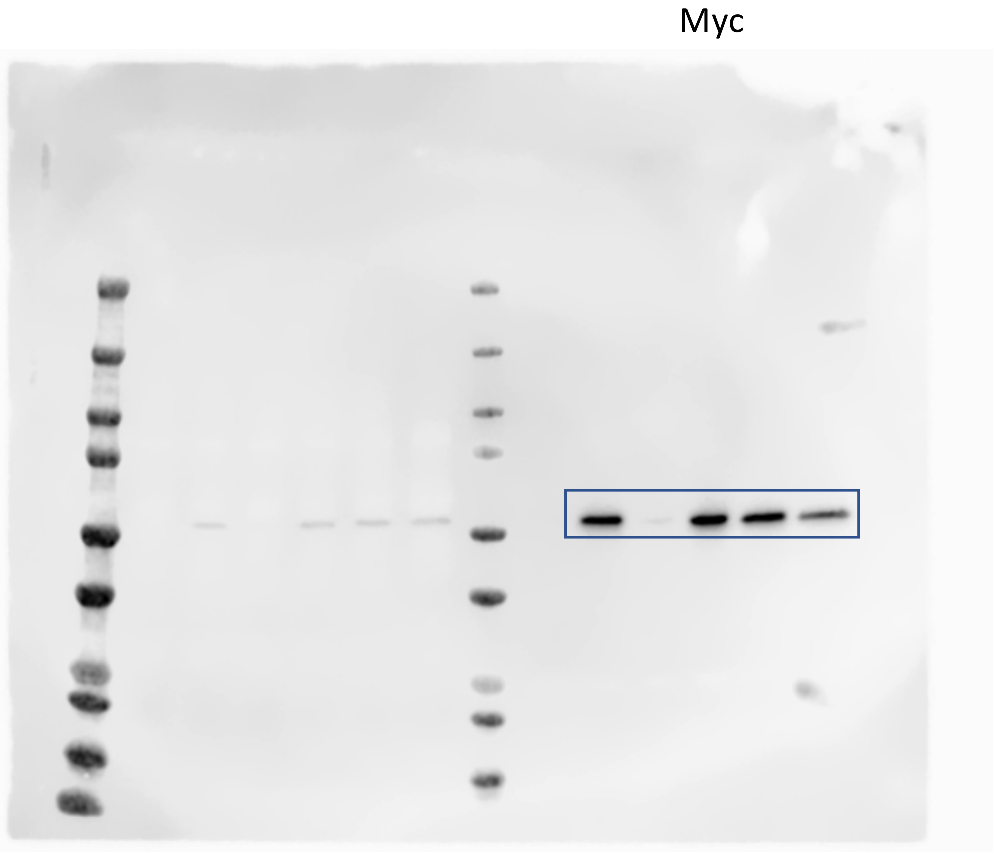

Supplement: Supplemental data [file jciinsight-6-138059-s046.pdf]
